# Pathway engineering in yeast for synthesizing the complex polyketide bikaverin

**DOI:** 10.1038/s41467-020-19984-3

**Published:** 2020-12-03

**Authors:** Meng Zhao, Yu Zhao, Mingdong Yao, Hala Iqbal, Qi Hu, Hong Liu, Bin Qiao, Chun Li, Christine A. S. Skovbjerg, Jens Christian Nielsen, Jens Nielsen, Rasmus J. N. Frandsen, Yingjin Yuan, Jef D. Boeke

**Affiliations:** 1 Frontier Science Center for Synthetic Biology and Key Laboratory of Systems Bioengineering (Ministry of Education), School of Chemical Engineering and Technology, Tianjin University, 300072 Tianjin, PR China; 2 SynBio Research Platform, Collaborative Innovation Center of Chemical Science and Engineering (Tianjin), Tianjin University, 300072 Tianjin, PR China; 3 Institute for Systems Genetics and Department of Biochemistry and Molecular Pharmacology, NYU Langone Health, New York, NY 10016 USA; 4 Section for Synthetic Biology, Department of Biotechnology and Biomedicine, Technical University of Denmark, Søltofts Plads, Building 223, Kongens Lyngby, Denmark; 5 Department of Biology and Biological Engineering, Chalmers University of Technology, Gothenburg, Sweden; 6 Novo Nordisk Foundation Center for Biosustainability, Technical University of Denmark, Kongens Lyngby, Denmark; 7 Department of Biomedical Engineering, NYU Tandon School of Engineering, Brooklyn, NY USA

**Keywords:** Synthetic biology, Metabolic engineering, Natural product synthesis, Applied microbiology

## Abstract

Fungal polyketides display remarkable structural diversity and bioactivity, and therefore the biosynthesis and engineering of this large class of molecules is therapeutically significant. Here, we successfully recode, construct and characterize the biosynthetic pathway of bikaverin, a tetracyclic polyketide with antibiotic, antifungal and anticancer properties, in *S. cerevisiae*. We use a green fluorescent protein (GFP) mapping strategy to identify the low expression of Bik1 (polyketide synthase) as a major bottleneck step in the pathway, and a promoter exchange strategy is used to increase expression of Bik1 and bikaverin titer. Then, we use an enzyme-fusion strategy to directly couple the monooxygenase (Bik2) and methyltransferase (Bik3) to efficiently channel intermediates between modifying enzymes, leading to an improved titer of bikaverin at 202.75 mg/L with flask fermentation (273-fold higher than the initial titer). This study demonstrates that the biosynthesis of complex fungal polyketides can be established and efficiently engineered in *S. cerevisiae*, highlighting the potential for natural product synthesis and large-scale fermentation in yeast.

## Abstract (web summary)

Bikaverin is a fungal-derived tetracyclic polyketide with antibiotic, antifungal and anticancer properties. Here, the authors employ various pathway engineering strategies to achieve high level production of bikaverin in baker’s yeast.

## Introduction

Fungal polyketides represent a large group of structurally and functionally diverse natural products with clinically relevant medical properties, including antibiotic, antifungal, immunosuppression, and anticancer activities^1–4^. This diversity is biosynthetically generated by the combination of modifying enzymes, such as methyltransferases and oxygenases, and a multifunctional enzyme polyketide synthase (PKS) that condenses multiple carboxyl units in a highly regulated, iterative process involving initiation, elongation, cyclization, and release^5^. Because the resulting molecules are often excellent candidates for therapeutics, the metabolic engineering of fungal polyketides, including both their biosynthesis and large-scale fermentation, is of substantial interest.

*Saccharomyces cerevisiae* is one of the most widely used hosts for the metabolic engineering of natural products including antibiotics, terpenoids, cannabinoids, and opiates^6–9^. Despite the genetic tractability and scalability of metabolic engineering in yeast and a few examples of the successful biosynthesis of fungal polyketides in *S. cerevisiae*, the biosynthesis and large-scale fermentation of complex polyketides in yeast remains a challenge, due to the large size of the PKS biosynthetic enzymes, complicated biosynthetic pathways, and highly regulated nature of the process^10–14^.

**Fig. 1:**
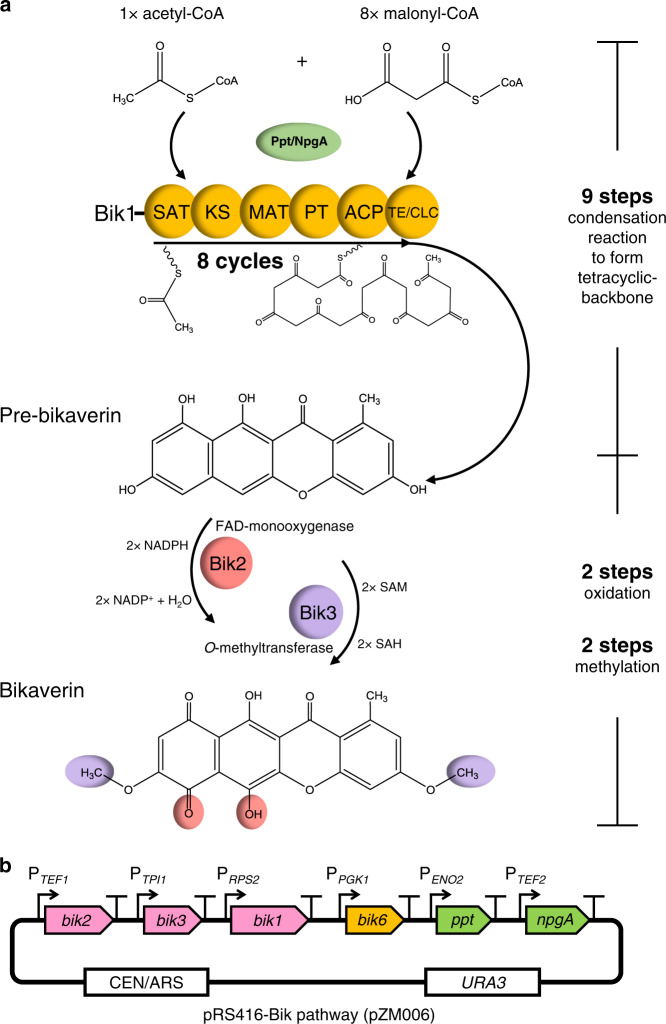
Bikaverin biosynthetic pathway in *S. cerevisiae*. **a** In the bikaverin biosynthetic pathway, the polyketide synthase Bik1, activated by phosphopantetheinyl transferase (PPTase), condenses one acetyl-CoA and eight malonyl-CoA units to form pre-bikaverin. The modifying enzymes Bik2 (monooxygenase) and Bik3 (*O*-methyl-transferase) convert pre-bikaverin into the final product bikaverin. The groups modified by Bik2 or Bik3 are highlighted in red or purple, respectively. SAT, starter unit acyltransferase; KS, ketosynthase; MAT, malonyl:ACP acyltransferase; PT, product template; ACP, acyl carrier protein; TE/CLC, thioesterase/claisen cyclase; SAM, S-adenosylmethionine; SAH, S-adenosylhomocysteine. **b** The constructed bikaverin pathway encoding plasmid. The *ppt1*, *npgA*, and bik genes (*bik1*, *bik2*, *bik3*, *bik6*) were codon-optimized, synthesized, and assembled into the pRS416 plasmid vector and transformed into *S. cerevisiae*.

Bikaverin is a red-colored tetracyclic polyketide with antibacterial and anticancer activities produced by members of the genus *Fusarium*^15–19^. A putative bikaverin biosynthetic gene cluster was identified in *Fusarium fujikuroi*, and a biosynthetic pathway was proposed based on knockout-based studies. However, the pathway has not been fully characterized nor verified via gain of function experiments. Among the six proteins encoded, enzymes Bik1, Bik2, and Bik3 are predicted to be responsible for the synthesis of bikaverin, while Bik4 and Bik5 are predicted transcription regulators, and Bik6 is a permease^18^. The polyketide carbon-skeleton of bikaverin is produced by the Type I PKS Bik1 (formerly called PKS4), which is a very large (over 2000 amino acid residues) multifunctional enzyme consisting of the following domains: starter unit acyltransferase (SAT), β-ketosynthase (KS), malonyl:ACP acyltransferase (MAT), product template (PT), acyl carrier protein (ACP), and thioesterase/claisen cyclase (TE/CLC)^20,21^. The SAT domain initially selects acetyl-CoA as a starting unit, and the KS and MAT domains condense eight units of malonyl-CoA to the growing polyketide chain, resulting in a polyketide chain, which is covalently tethered to the ACP domain throughout chain elongation. The PT and TE/CLC domains control and cyclize the chain, releasing the intermediate pre-bikaverin from Bik1. Pre-bikaverin is modified with two oxidations catalyzed by the FAD-dependent monooxygenase Bik2, and two methylations catalyzed by *O*-methyltransferase Bik3, to form the final product bikaverin (Fig. 1a). To expand the metabolic engineering of polyketides in yeast, the bikaverin pathway is an example of constructing and characterizing a cell factory in *S. cerevisiae* for the production of this fungal polyketide.

In this work, we successfully recode, construct, and characterize the biosynthetic pathway of the complex polyketide bikaverin in *S. cerevisiae*. We develop a GFP-mapping method for quickly identifying the expression of *bik1* as the bottleneck in the pathway and use a promoter exchange strategy to increase expression of *bik1* and subsequently, bikaverin titer. In addition, we verify the genes essential for bikaverin synthesis and characterize the biosynthetic pathway through a bottom-up strategy. Finally, we optimize bikaverin production by developing an enzyme-fusion method to couple the modifying enzymes Bik2 and Bik3 to create a short substrate channel between the enzymes, achieving a ~270-fold increase in the final titer of the molecule.

## Results

### Establishing the biosynthesis of bikaverin in *S. cerevisiae*

To heterologously express the bikaverin biosynthetic pathway in *S. cerevisiae*, we designed a yeast-recoded bikaverin gene cluster comprising the genes *bik1*, *bik2*, *bik3*, and *bik6*, as well as the coding genes of phosphopantetheinyl transferase (PPTase) needed for post-translational activation of the ACP domain in Bik1. We retrieved the protein sequences of Bik1, Bik2, Bik3, and Bik6 from the NCBI Protein database: (accession numbers: Bik1, S0DZM7; Bik2, S0E2X6; Bik3, S0E608; Bik6, S0DZN4)^22^. Protein sequences were reverse-translated and codon-optimized for expression in *S. cerevisiae*. Then, we synthesized these *bik1*, *bik2*, *bik3*, and *bik6* genes under the control of yeast endogenous promoters, P_*RPS2*_, P_*TEF1*_, P_*TPI1*_, and P_*PGK1*_, respectively (Fig. 1b). In addition, to facilitate the post-translational activation of the PKS, we chose two PPTases to activate Bik1: Ppt1 (accession number CCE73639), the native PPTase from *F. fujikuroi*, and NpgA (accession number AAF12814), a PPTase from *A. nidulans*, which has previously been successfully used in yeast to activate PKSs and nonribosomal peptide synthetases (NPRSs)^6,12,13^. Both PPTase genes were added into the constructed yeast bikaverin expression cassette under the control of P_*ENO2*_ and P_*TEF2*_ promoters, respectively. The resulting bikaverin expression cassette was transformed into yeast, as strain yZM006. This strain, however, failed to turn red, and no bikaverin was detected (Supplementary Fig. 1).

**Fig. 2:**
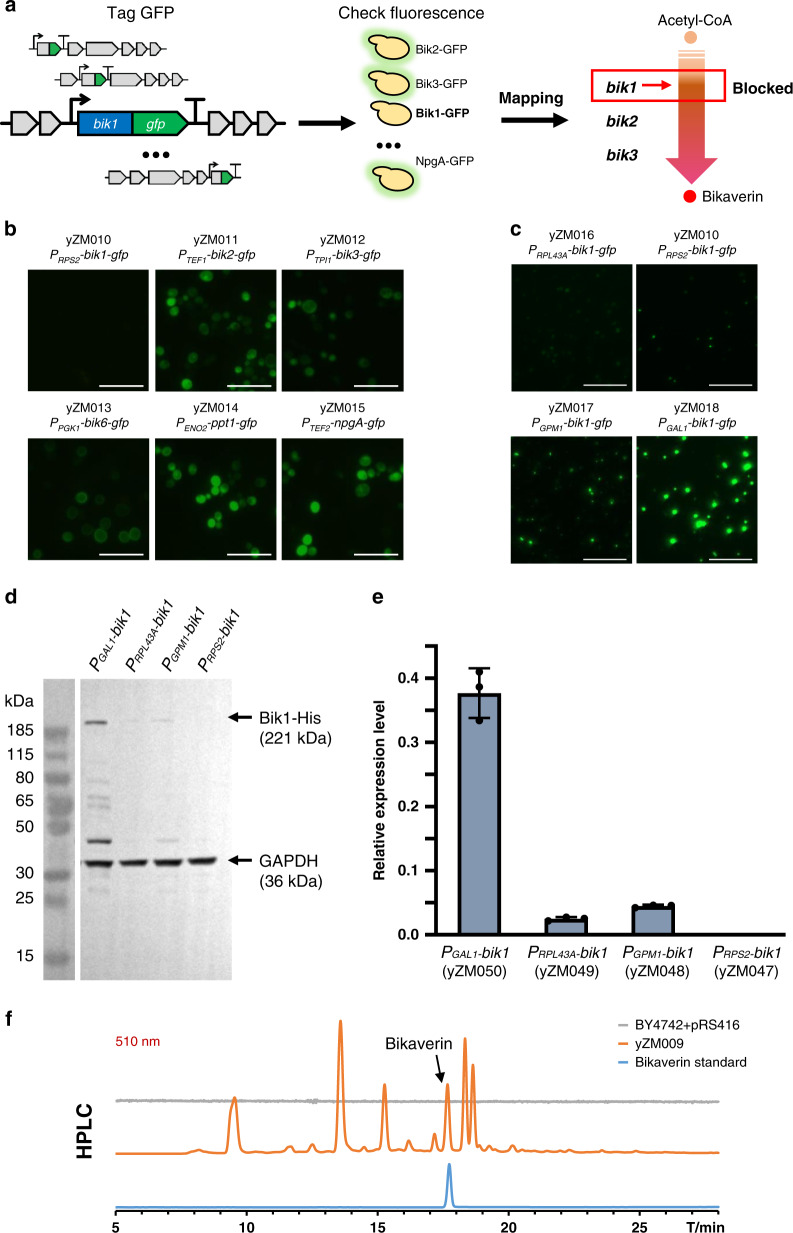
Identifying bottlenecks in the bikaverin pathway. **a** GFP-mapping strategy to identify bottlenecks. Each bikaverin pathway gene (in gray) was tagged with GFPtag (in green) separately to check their expression and Bik1 was identified as the bottleneck in the pathway. **b** The results of fluorescence checking in GFP-mapping strategy. Scale bar = 20 µm. **c** Fluorescence of Bik1-GFP using different promoters. Scale bar = 50 µm. **d** Western blots to check expression of C-terminally His-tagged Bik1 using different promoters. GAPDH was used as the internal reference protein. The original uncropped source image is provided in Supplementary Fig. 12. **e** Quantitative relative expression level of Bik1 using these promoters. Relative expression level was the intensity of Bik1 band divided by the intensity of internal reference GAPDH band. Data are presented here as mean values ± standard deviation (SD) calculated from *n* = 3 biological replicates. **f** The production of bikaverin in strain yZM009 was confirmed by HPLC analysis. Experiments in **b**–**d** were repeated three times independently with similar results. Source data underlying Fig. 2e are provided as a Source Data file.

To probe the bottlenecks limiting biosynthesis in the bikaverin pathway, we developed a GFP-mapping strategy to confirm that all genes were well expressed (Fig. 2a). The coding sequence for green fluorescent protein (GFP) was fused in frame to the 3′ end of the individual genes. Fluorescent microscopy showed a strong GFP signal in cells where the genes *bik2*, *bik3*, *bik6*, *ppt1*, or *npgA* were tagged, whereas cells meant to express *bik1* displayed a weak signal, suggesting that the Bik1 level was limiting bikaverin biosynthesis (Fig. 2b). Bik1 is a large and multidomain PKS enzyme, roughly 220 kDa in size. We hypothesized that the protein was either poorly expressed from our construct or degraded in yeast because of its large size. To test this hypothesis, we replaced the initial promoter P_*RPS2*_ with several different yeast endogenous promoters: P_*RPL43A*_, P_*GPM1*_, and P_*GAL1*_. It was observed that the Bik1-GFP was strongly expressed only when driven by the galactose-inducible *GAL1* promoter (strain yZM018) (Fig. 2c). Consistent with this result, only strain yZM009 turned red, and only when grown on galactose medium (Supplementary Fig. 1). In order to further confirm Bik1 expression, we tagged Bik1 with poly-histidine and checked the production and degradation of the protein by western blot. Expression of Bik1 was much higher when it was driven by P_*GAL1*_ compared to other promoters, with partial protein degradation detected (Fig. 2d, e). Finally, bikaverin synthesized was also confirmed by HPLC and MS (Fig. 2f and Supplementary Fig. 2).

**Fig. 3:**
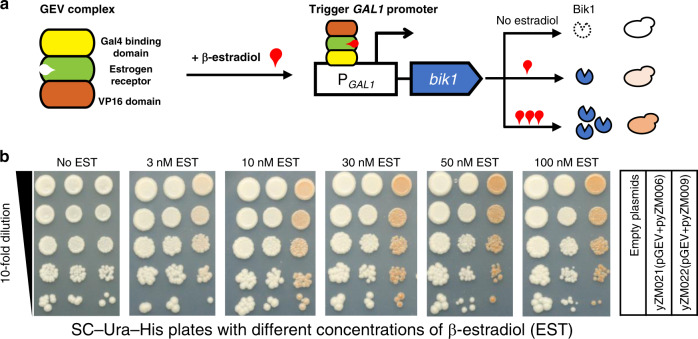
Using the GEV system to regulate Bik1 expression and its effect on bikaverin synthesis. **a** Working principle diagram of GEV system. The GEV inducible system regulates the *GAL1* promoter to express Bik1 by β-estradiol (EST). In this system, GEV–EST complex will activate transcription from the *GAL1* promoter and increasing the concentration of β-estradiol in the glucose medium leads to stronger expression. **b** Using GEV system to regulate Bik1 expression. Spot assays were performed to check bikaverin production on SC-Ura-His plates with a gradient of estradiol concentration. The empty pRS413 and pRS416 plasmids were co-transformed into BY4741 as the empty control. The strains yZM006 (with *P*_*RPS2*_*-bik1*) and yZM009 (with *P*_*GAL1*_*-bik1*) were transformed with the GEV plasmid, generating strains yZM021 and yZM022, respectively.

To further confirm that bikaverin biosynthesis was the consequence of higher expression of Bik1, and not because of the use of galactose instead of glucose as the carbon source, we introduced a galactose-independent system to regulate the *GAL1* promoter (Fig. 3a). In this system, a GAL4(1-93).ER.VP16 (GEV) plasmid is expressed to produce a tribrid protein comprising a Gal4 DNA-binding domain, an estrogen receptor and a VP16 domain^23,24^. This complex, when bound to β-estradiol (EST), will translocate into the nucleus and bind to P_*GAL1*_ to activate downstream gene transcription, and an increase in EST availability will lead to higher rates of transcription^23,24^. With *bik1* under the control of this system, higher concentrations of EST in the glucose medium resulted in higher expression of Bik1 and deeper red colony color (Fig. 3b). This confirmed that the production of bikaverin indeed resulted from promoter exchange to P_*GAL1*_ and the subsequent higher expression of *bik1*.

### Characterization of the bikaverin pathway in *S. cerevisiae*

**Fig. 4:**
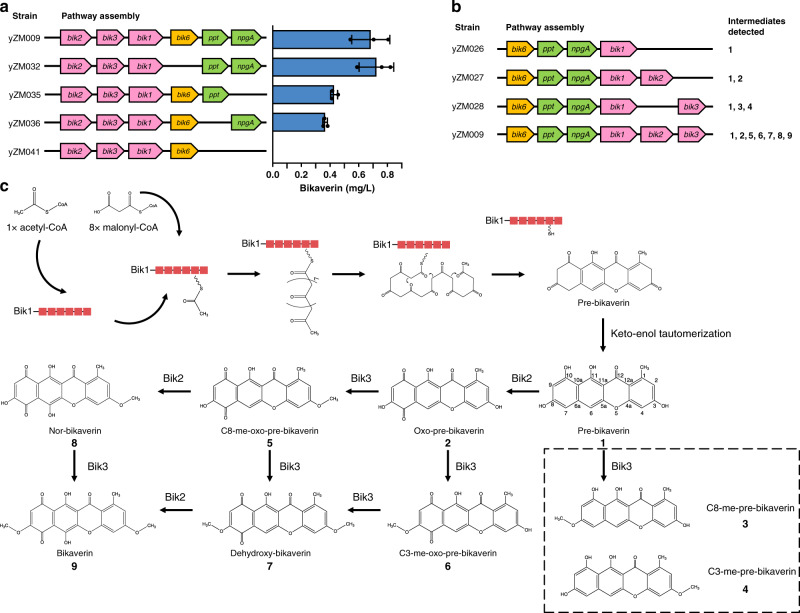
Characterization of bikaverin synthesis in yeast. **a** A top-down strategy was used to measure the effects of *bik6*, *ppt1*, and/or *npgA* deletion on bikaverin production. Data are presented here as mean values ± SD calculated from *n* = 3 biological replicates. **b** As a bottom-up strategy, pathways containing different combinations of *bik1*, *bik2*, and *bik3* were constructed. The intermediates (**1**–**9** as in **c**) was detected by HPLC–MS (Supplementary Figs. 5–8) and used to decipher the biosynthetic pathway of bikaverin. **c** The putative bikaverin biosynthesis pathway in *S. cerevisiae*. Multi-domain protein Bik1 is displayed as a series of connected red rectangles. Boxed intermediates were only detected in the absence of *bik2* (Supplementary Fig. 6). Source data underlying **a** are provided as a Source Data file.

With bikaverin biosynthesis successfully established in *S. cerevisiae*, we turned to the characterization of this biosynthetic pathway using both top-down and bottom-up strategies. To probe the essentiality of *bik6* for bikaverin production in yeast and whether both PPTases can activate Bik1, we used a top-down approach to delete *bik6*, *ppt1*, and *npgA* separately or in combination, from the yeast-recoded bikaverin pathway. Unlike in *F. fujikoroi*, where deletion of *bik6* was reported to reduce bikaverin yield, deletion of *bik6* in *S. cerevisiae* had minimal effect on bikaverin yield (Fig. 4a and Supplementary Fig. 9). Conversely, when we deleted both *ppt1* and *npgA*, bikaverin was not synthesized at all, suggesting there was no intrinsic PPTase in yeast that could activate Bik1 by phosphopantetheinylation, whereas deletion of either *ppt1* or *npgA* reduced but did not eliminate bikaverin titers compared to the original strain. The fact that bikaverin was still successfully synthesized in both cases indicated that both Ppt1 and NpgA were capable of activating the ACP domain of Bik1. Interestingly, strains containing two copies of *ppt1* or *npgA* showed a different result: doubling *ppt1* did not show improvement in bikaverin titers compared to single copy of *ppt1*. But doubling *npgA* improved the bikaverin production to a similar level as the original design containing both *ppt1* and *npgA* (Supplementary Fig. 3). This result suggests that NpgA may be a more effective PPTase for activating the ACP domain of Bik1 in yeast.

To further characterize bikaverin biosynthesis and establish the order of reactions in this pathway, we used a bottom-up strategy to build constructs with different combinations of *bik1*, *bik2*, and *bik3* and analyzed the resulting intermediates using HPLC–MS^25^ (Fig. 4b). As expected, pre-bikaverin (*m/z* 325.06) (**1**) was detected in all strains containing *bik1* (Fig. 4b and Supplementary Fig. 5). Oxo-pre-bikaverin (*m/z* 339.04) (**2**), the expected intermediate resulting from the C7 oxidation of pre-bikaverin by the monooxygenase Bik2, was detected in strains containing *bik2* (yZM027 and yZM009). Interestingly, di-nor-bikaverin, the predicted product from the further oxidation of oxo-pre-bikaverin, was not detected in either strain expressing Bik2 (yZM027 and yZM009), indicating that in yeast the monooxygenase cannot oxidize the C6 position without prior oxidation of the C7 position (Supplementary Fig. 6).

In the strain containing only *bik1* and *bik3* (yZM028), two small peaks of *m/z* 339.09 and 339.08 were detected (Supplementary Fig. 7). HPLC–MS retention time confirmed that neither peak was oxo-pre-bikaverin (**2**), and neither peak was detected in strains that are lacking *bik3*. We speculated that these peaks are intermediates resulting from Bik3-catalyzed methylation of pre-bikaverin to form C8-me-pre-bikaverin (**3**) and C3-me-pre-bikaverin (**4**). These two peaks were small and not detected in strain yZM009 containing the entire pathway, suggesting that pre-bikaverin can be methylated by Bik3 but that this substrate may not be preferred by Bik3, compared to oxo-pre-bikaverin (**2**) and other downstream substrates including C3/C8 me-oxo-pre-bikaverin (**6/5**) and nor-bikaverin (**8**).

In strain yZM009 containing all the pathway genes, pre-bikaverin (**1**) and oxo-pre-bikaverin (**2**) were detected (Supplementary Fig. 8a and b). In addition, two peaks of *m/z* 353.05 were detected (Supplementary Fig. 8c); these were postulated to be the two mono-methylated forms of oxo-pre-bikaverin: C3-me-oxo-pre-bikaverin (**6**) and C8-me-oxo-pre-bikaverin (**5**). In addition, a peak corresponding to dehydroxy-bikaverin (*m/z* 367.07) (**7**), the di-methylated form of oxo-pre-bikaverin, was detected in this strain (Supplementary Fig. 8d), suggesting that Bik3 is able to methylate oxo-pre-bikaverin twice in yeast. Nor-bikaverin (**8**), the intermediate produced from the further oxidation of C8-me-oxo-pre-bikaverin (**5**) by Bik2, was also detected (*m/z* 369.05) and confirmed by comparison to the previously published MS trace of the molecule^25^. However, at *m/z* 369.05, only one peak of nor-bikaverin was detected, which suggested that C8-me-oxo-pre-bikaverin, not C3-me-oxo-pre-bikaverin, was the favored substrate of *bik2*, although it is possible that the oxidized form of C3-me-oxo-pre-bikaverin cannot be isolated using the HPLC–MS conditions tested (Supplementary Fig. 8e). With this certain preference for substrates, Bik2 can catalyze the oxidation of C8-me-oxo-pre-bikaverin, while Bik3 can methylate both C3-me-oxo-pre-bikaverin and C8-me-oxo-pre-bikaverin in *S. cerevisiae*. The final product, bikaverin (*m/z* 383.06) (**9**), was only detected in the strain containing the complete pathway, confirming that Bik1, Bik2, and Bik3 are all essential and sufficient for the de novo biosynthesis of bikaverin (Supplementary Figs. 4 and 8f).

Based on these results, we mapped out the putative bikaverin biosynthetic pathway in yeast (Fig. 4c). Pre-bikaverin is first oxidized to oxo-pre-bikaverin by Bik2, then to dehydroxy-bikaverin by two methylation steps catalyzed by Bik3. Dehydroxy-bikaverin can then be oxidized by Bik2 again to form the final product bikaverin. In addition, the intermediate C8-me-oxo-pre-bikaverin, but not C3-me-oxo-pre-bikaverin, can also be oxidized by Bik2 to nor-bikaverin and then methylated by Bik3 to form bikaverin.

### Pathway optimization to increase bikaverin production

**Fig. 5:**
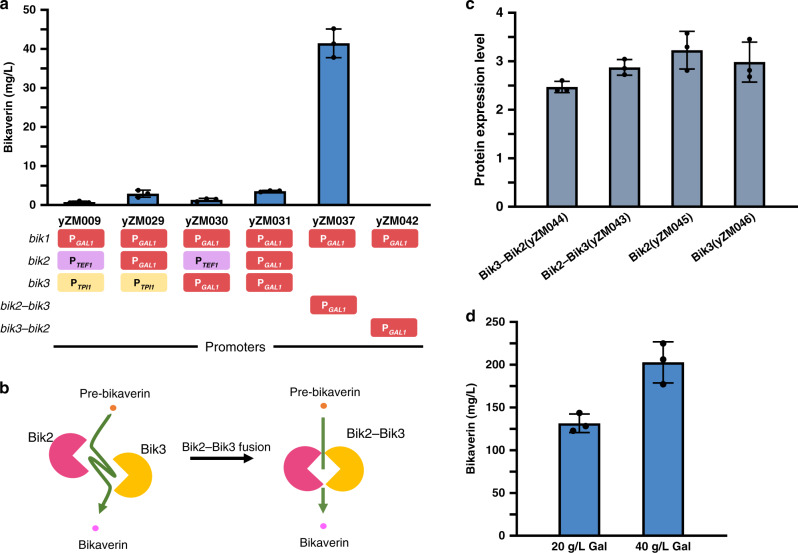
Optimization to improve bikaverin titer using promoter exchange and enzyme fusion strategies. **a** Driving *bik2* and *bik3* using P_*GAL1*_ led to a modest increase (yZM031). Promoters used are shown in different color rectangles. Fusing Bik2 and Bik3 led to a dramatic increase in bikaverin titer (yZM037), 60-fold higher than the original strain (yZM009). The strain with the ‘opposite polarity’ fusion as Bik3–Bik2 produced no bikaverin (yZM042). **b** Substrates channel model between Bik2 and Bik3. The fusion of Bik2 and Bik3 may form a short and adaptive substrate channel. **c** Relative protein expression levels of Bik2–His6, Bik3–His6, fusion proteins Bik3–Bik2–His6 and Bik2–Bik3–His6 using P_*GAL1*_ promoter by quantitative western blotting. Each protein was fused with 6×His-tag at its C-terminus. Protein expression level was measured as the intensity of target protein band divided by the intensity of the GAPDH band. **d** The bikaverin titer from flask fermentation. Strain yZM037 was cultured in YP medium supplemented with 20 or 40 g/L galactose as the carbon source. Data in **a**, **c**, **d** are presented as mean values ± SD from *n* = 3 biological replicates. Source data underlying **a**, **c**, and **d** are provided as a Source Data file.

Although the biosynthetic pathway of bikaverin was reconstituted in *S. cerevisiae*, titers of the molecule remained low. To further optimize the pathway in yeast, we applied the promoter exchange strategy described earlier to modifying enzymes in the bikaverin pathway. It was found that changing the promoter of *bik2* or *bik3* to P_*GAL1*_ resulted in an increase in bikaverin titer from ~0.74 to ~2.90 mg/L or to ~1.37 mg/L, respectively (Fig. 5a). Further, changing both *bik2* and *bik3* promoters to P_*GAL1*_ resulted in a bikaverin titer of ~3.65 mg/L, as in strain yZM031, which was five-fold higher than the original strain yZM009.

In addition, characterization of the bikaverin biosynthetic pathway highlighted that pre-bikaverin is modified in a stepwise manner by the monooxygenase Bik2 and the *O*-methyltransferase Bik3. Interestingly, substrates need to shuttle repeatedly between Bik2 and Bik3 to convert oxo-pre-bikaverin to bikaverin. We hypothesized that repeated substrate-shuttling may limit the synergistic catalytic efficiency of Bik2 and Bik3. Thus, we reasoned that we could increase the catalytic efficiency of Bik2 and Bik3 by fusing them to form a short and adaptive substrate channel for simplifying substrate transfer between these two enzymes and preventing the diffusion of undesirable complex substrates.

To test this, we first built the fusion protein of Bik2 and Bik3 (Bik2–Bik3) with a (GGGS)_3_ linker connecting the C-terminus of Bik2 to the N-terminus of Bik3. As a result, the titer of bikaverin significantly increased to ~41.4 mg/L in the strain yZM037 containing Bik2–Bik3, ~11-fold higher than that in the strain yZM031 with separate Bik2 and Bik3, and nearly 60-fold higher than that in the original strain producing bikaverin (yZM009) (Fig. 5a). Conversely, the pathway using the reverse fusion protein connecting the N-terminus of Bik2 to the C-terminus of Bik3 (Bik3–Bik2) in strain yZM042 showed no production of bikaverin (Fig. 5a). To understand these two results, we modeled the protein structures of the two Bik2–Bik3 fusion constructs. The active site and FAD-binding pocket of Bik2 are near its N-terminal, while the active site and SAM-binding pocket of Bik3 are close to C-terminal (Supplementary Fig. 10). The fusion of Bik3–Bik2 is postulated to disturb the active pocket structures of Bik2 and Bik3. This could be the reason that no detectable bikaverin was produced in the Bik3–Bik2 strain. To check the effect of protein fusion on the stability of the fusion proteins, we tagged Bik2, Bik3, Bik2–Bik3, and Bik3–Bik2 with poly-His tags and checked expression levels using western blots (Supplementary Fig. 11). We found that the expression of Bik2–Bik3 and Bik3–Bik2 was similar to Bik2 or Bik3, with both fusion proteins expressed at slightly lower levels (Fig. 5c). This confirmed that the improved titers of bikaverin seen were due to the fusion of Bik2–Bik3, and not expression level or protein stability change. We speculate that the Bik2–Bik3 fusion leads to a steric effect in which a substrate-shuttle channel between Bik2 and Bik3 allows substrates to transfer efficiently between Bik2 and Bik3, resulting in improved bikaverin titers.

In order to further improve the titer, we changed the fermentation medium from synthetic medium (SC–Ura) to rich medium (YP + galactose). Bikaverin titer rose to 131.48 mg/L when yZM037 was cultured in YP medium with 20 g/L galactose as carbon source. With galactose concentration increased to 40 g/L, the titer was further improved to 202.75 mg/L, about a 273-fold increase compared to the original titer (Fig. 5d).

## Discussion

Fungal polyketides display remarkable structural and functional diversity and are excellent candidates for the development and biosynthetic engineering of new therapeutics with anticancer, antifungal, antibiotic, and immunomodulation properties. In this study, we demonstrated the efficient biosynthesis of the complex fungal polyketide bikaverin in the heterologous host *S. cerevisiae* using synthetic biology approaches. To achieve this, we reconstructed the bikaverin pathway in yeast by recoding and synthesizing the genes *bik1*, *bik2*, *bik3*, and *bik6* from *F. fujikoroi* along with two PPTases to activate the ACP domain of the PKS Bik1.

Compared to bacteria, yeast is a better model organism for biosynthetic engineering of fungal polyketides, with many available synthetic biological tools including pathway assembly, CRISPR genome editing and even whole chromosome synthesis, and SCRaMbLE^26–32^. Using these tools available in yeast, we were able to characterize the bikaverin biosynthetic pathway using top-down and bottom-up strategies. Our result showed that genes *bik1*, *bik2*, and *bik3* are essential for the production of bikaverin, while in yeast, omitting the permease encoding gene *bik6* had minimal effect. We also successfully confirmed the production of the proposed intermediates in the bikaverin biosynthetic pathway and determined the reactions catalyzed by Bik2 and Bik3. In addition, the use of yeast promoters to drive the expression of individual bikaverin pathway genes led us to establish a system that are independent of pre-existing transcription regulatory mechanisms in *F. fujikuroi*. In summary, our strains display stable, tunable, and scalable bikaverin production unaffected by environmental conditions, an added advantage of using yeast as a host for polyketide bioengineering. Finally, we were able to achieve titers of more than 200 mg/L using flask fermentation without modifying any yeast endogenous genes.

By establishing and optimizing the bikaverin pathway, we have demonstrated effective and practical methods for the metabolic engineering of polyketides in yeast. Establishing an effective metabolic pathway is the first step for the metabolic engineering of heterologous expression. It is common that reconstituted pathways do not produce the desired molecule in heterologous hosts, and it is often difficult to figure out the bottleneck step. In our study, a GFP-mapping strategy helped us to identify Bik1 as the initial bottleneck step quickly and efficiently. Based on this result, we debugged the polyketide pathway and further improved the bikaverin titer by testing several promoters and using the strong, inducible promoter P_*GAL1*_ finally. This method can be used to figure out bottlenecks in many other metabolic pathways. In addition, we used an enzyme-fusion strategy to create a substrate channel between two modifying enzymes. We postulated that this fusion may help channel substrates from one modifying enzyme to the other, minimizing diffusion of intermediates and improving efficiency of substrate transfer between the two enzymes. In other biosynthetic pathways with modifications, such as methylation, glycosylation, and hydroxylation that employ similar shuttling, this protein fusion strategy might be key to improving product titers^33–36^.

In conclusion, we have demonstrated the potential of *S. cerevisiae* to synthesize complex polyketides in this study. With more and more PKS successfully expressed in yeast, we anticipate the expansion of metabolic engineering of PKS in *S. cerevisiae*, including the development of methods in yeast, such as domain swapping to generate diverse polyketides. It has been reported that swapping domains among different PKSs results in diverse functions in vitro^37–39^. We hope that our study, as well as other pre-existing synthetic biology tools, will result in the use of *S. cerevisiae* as a robust platform to explore the chemical development of polyketides and therapeutic applications with their diverse bioactivities.

## Methods

### Yeast strains and plasmid assembly

All strains, plasmids used in this study were listed in Supplementary Tables 1 and 2. The protein sequences of Bik1, Bik2, Bik3, Bik6, Ppt1, and NpgA were obtained from the NCBI database (accession numbers: Bik1, S0DZM7; Bik2, S0E2X6; Bik3, S0E608; Bik6, S0DZN4; Ppt1, CCE73639; NpgA, AAF12814). Protein sequences were reverse-translated into nucleotide sequences and codon optimized for expression in yeast using online tool BioPartsBuilder^40^. The recoded DNA sequence is provided in Supplementary Data 1. Yeast promoters and terminators were PCR amplified from BY4742 genomic DNA. All pathway genes (*bik1*, *bik2*, *bik3*, *bik6*, *ppt1*, *npgA*) were individually constructed as transcription units (TUs) consisting of promoter, coding sequence, and terminator employing the yeast Golden Gate (yGG) DNA assembly method^41^. We used the versatile genetic assembly system (VEGAS) to assemble all the TUs into pRS416 plasmid backbone in BY4742 (*MAT*α *ura3∆0 leu1∆0 lys2∆0 his3∆1*)^42,43^. The final construct was miniprepped from yeast and transformed into *E. coli* for sequencing verification and storage. The correct plasmid with the complete bikaverin pathway was re-transformed into new BY4742 strains for further study.

Yeast strains were grown using YPD-rich medium or defined SC media with appropriate components dropped out or compounds added as indicated. All yeast transformations in this study were performed with standard lithium acetate protocols^43^.

### Fusion protein construction in GFP-mapping strategy and western blot

To add a GFP tag to the C-terminal of each coding sequence, the GFP-KanMX6 fragments were PCR amplified with corresponding homology arms. Plasmid pFA6a-GFP(S65T)-kanMX6 was used as PCR template and the primers used are listed in Supplementary Table 3. Then the PCR amplicon was transformed into the yeast strain already carrying the complete bikaverin pathway. Successful integration was selected on YPD + G418 plates and confirmed by colony PCR and Sanger sequencing.

To add a 6 × His tag to the C-terminal of each coding sequence, the 6 × His-KanMX6 fragment was PCR amplified from plasmid pFA6a-GFP(S65T)-kanMX6 with primer His-tag-F and His-tag-R at first. Then the first round PCR amplicon was used as PCR template for amplifying the fragments added with corresponding homology arms. The corresponding primers are shown in Supplementary Table 3. Then the second round PCR amplicon was transformed into the yeast strain as above. And successful integration was selected on YPD + G418 plates and confirmed by colony PCR and Sanger sequencing too.

### Fluorescence microscopy

Pictures were taken using an EVOS-FL Auto cell imaging system (Invitrogen) with the ×20 objective lens. Yeast cells were cultured on plates using appropriate selective media for 2 days, then picked onto wet mount slides for visualization.

### Strain fermentation and bikaverin extraction

Yeast strains were cultured in 5 mL of SC–Ura liquid medium at 30 °C, overnight to saturation. Cells were harvested by centrifugation at 1600×*g* for 5 min, followed by 3 × 2 mL washes with water, then inoculated to an initial *A*_600_ of 0.5 in 30 mL of SC–Ura liquid medium with either glucose or galactose as the carbon source. Cultures were incubated at 30 °C for 96 h. Appropriate volumes of cell cultures (2 mL for Bik2–Bik3 fusion strains, 10 mL for other strains) were centrifuged for 10 min at 13,800×*g*. Cell pellets were resuspended with 0.5 mL water in a 2 mL tube, and 200 µL of acid-washed glass beads (Sigma, G8772-100G) were added into the tubes along with 0.5 mL ethyl acetate and 0.5 µL formic acid. Resulting suspensions were vortexed for 20 min and centrifuged for 10 min at 13,800×*g* before the supernatant was transferred to a new tube; this was repeated for five times. Finally, extracts were dissolved in 500 µL acetonitrile (ACN) and centrifuged for three times at 13,800×*g*, 10 min. Resulting extracts were used for HPLC and HPLC–MS analysis.

### HPLC analysis

HPLC analysis of bikaverin was performed on a Waters 2695 system equipped with a 2489 UV detector operating at 510 nm. Separation was achieved using a HyPURITY C18 5 μm 150 × 4.6 mm column (Thermo Fisher, 22105-154630) at 25 °C. The HPLC program was as follows: ACN as solvent A and 0.1% formic acid as solvent B; flow rate 0.8 mL/min; injection volume 20 µL; 0 min 30%A/70%B, 3 min 30%A/70%B, 5 min 35%A/65%B, 20 min 65%A/35%B, 25 min 80%A/20%B, 30 min 80%A/20%B, 30.5 min 30%A/70%B, and 45 min 30%A/70%B. Bikaverin standards (Sigma, SML0724) at the following concentrations: 60, 30, 15, 7.5, 3.75, 1.87 µg/mL, were used to establish a standard curve to calculate yields.

### LC/ESI–MS analysis

All MS data was measured by a HPLC/ESI–MS method. Bruker micrOTOF-Q II instrument connected with Agilent HPLC system (Agilent G1312B SL binary; Agilent G1367C SL WP) were used to detect bikaverin and pathway intermediates. The HPLC program and column used here were the same as the method used in HPLC analysis. Electrospray ionization (ESI) mass spectrometry was performed in positive mode and set as follows: voltage set capillary at 4500 V, set end plate offset at −500 V; nebulizer 2.0 bar; dry heater 180 °C; dry gas 6.0 l/min. The mass data was analyzed using Compass Data Analysis software. Water Empower 3.0 software was used to analyze chromatography data.

### Western blot

To quantify protein expression by western blot, a polyhistidine-tag (6 × His-tag) was attached to C terminals of each protein. Strains were cultured in SC–Ura medium with the appropriate carbon source at 30 °C for 20 h. Then 10 *A*_600_ units of yeast cell were harvested, washed by water and resuspended by 500 μl of 0.3 M NaOH. After incubation at room temperature for 5 min, cells were washed with 500 μl water, spun down and resuspended in 100 μl lysis buffer (4% β-mercaptoethanol, 0.06 M Tris–HCl, pH 6.8, 2% SDS, 5% glycerol), boiled for 10 min and centrifuged again at highest speed (14,000×*g*). After protein quantification by Bradford method, 10 μl supernatant (containing ~40 μg total protein) was loaded per lane of 12% SDS–PAGE gel. Proteins were transferred to a PVDF membrane and blocked by 5% BSA buffer. Anti-His antibody (1:2000, TransGen HT501, China) and anti-GAPDH antibody (1:2000, TransGen HC301, China) were used to probe target proteins and internal reference protein GADPH. Goat anti-mouse IgG (H + L) HRP-conjugated antibody (1:3000, TransGen HS201, China) was used as the secondary antibody. Multi-Chemiluminescent Blot Imaging System (Tanon-4800, China) was used for image collection. ImageJ software was used to quantify the intensities of protein bands (https://imagej.nih.gov/ij/).

### Homology modeling and structural analysis in silico

To explore the effect of protein fusion Bik2 and Bik3, the structure models of Bik2, Bik3 were built and optimized using EasyModeller (Version 4.0)^44^. The high-resolution complex structures of rifampicin monooxygenase with FAD (PDB ID: 5KOW) and methyltransferase with SAM (PDB ID: 5w7p-A) were used as the templates of Bik2 and Bik3, respectively. Modeled structures were analyzed using Pymol software (Version 1.7)^45^.

### Reporting summary

Further information on research design is available in the Nature Research Reporting Summary linked to this article.

## Supplementary information

Supplementary Information

Peer Review File

Description of Additional Supplementary Files

Supplementary Data 1

Reporting Summary

## Data Availability

Data supporting the findings of this work are available within the paper and its Supplementary Information files. A reporting summary for this Article is available as a Supplementary Information file. The datasets and materials generated and analyzed during the current study are available from the corresponding author upon request. The protein sequences of Bik1, Bik2, Bik3, Bik6, Ppt1, and NpgA were obtained from the NCBI Protein database (accession numbers: Bik1, S0DZM7; Bik2, S0E2X6; Bik3, S0E608; Bik6, S0DZN4; Ppt1, CCE73639; NpgA, AAF12814). Their DNA sequences used in this study were provided in Supplementary Data 1. The templates of Bik2 and Bik3 used for homology modeling were obtained from RCBS-PDB database (PDB ID: 5KOW and 5W7P-A, respectively). The LC–MS spectra data are available in Supplementary Figs. 4–8.  Source data are provided with this paper.

## Source data

Source Data
