# Supplementary material for: Pathway engineering in yeast for synthesizing the complex polyketide bikaverin
Source: Nat Commun. 2020 Dec 3;11:6197. doi: 10.1038/s41467-020-19984-3 (PMC7713123; doi:10.1038/s41467-020-19984-3)
Supplement: Supplementary file 1 — Supplementary Information [file 41467_2020_19984_MOESM1_ESM.pdf]

Pathway engineering in yeast for synthesizing the complex polyketide bikaverin

Zhao *et al.*

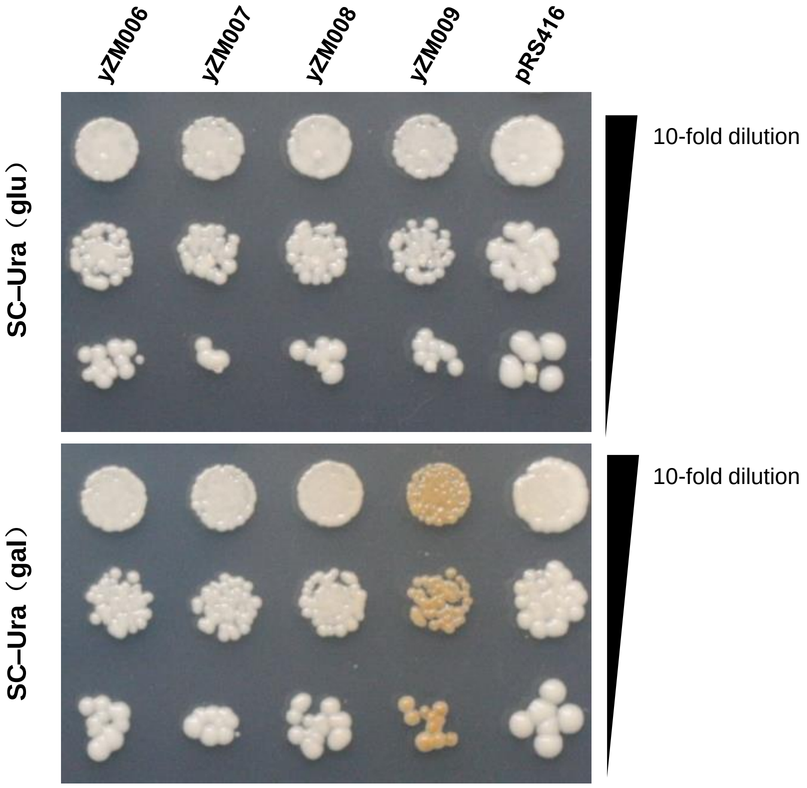

Supplementary Fig. 1. Phenotypes of bikaverin pathways with *bik1* driven by different promoters, growing on SC–Ura plate with glucose or galactose as carbon source for 2 days. yZM006, P_{RPS2}-*bik1*; yZM007, P_{RPL43A}-*bik1*; yZM008, P_{GPM1}-*bik1*; yZM009, P_{GAL1}-*bik1*; pRS416, empty control.

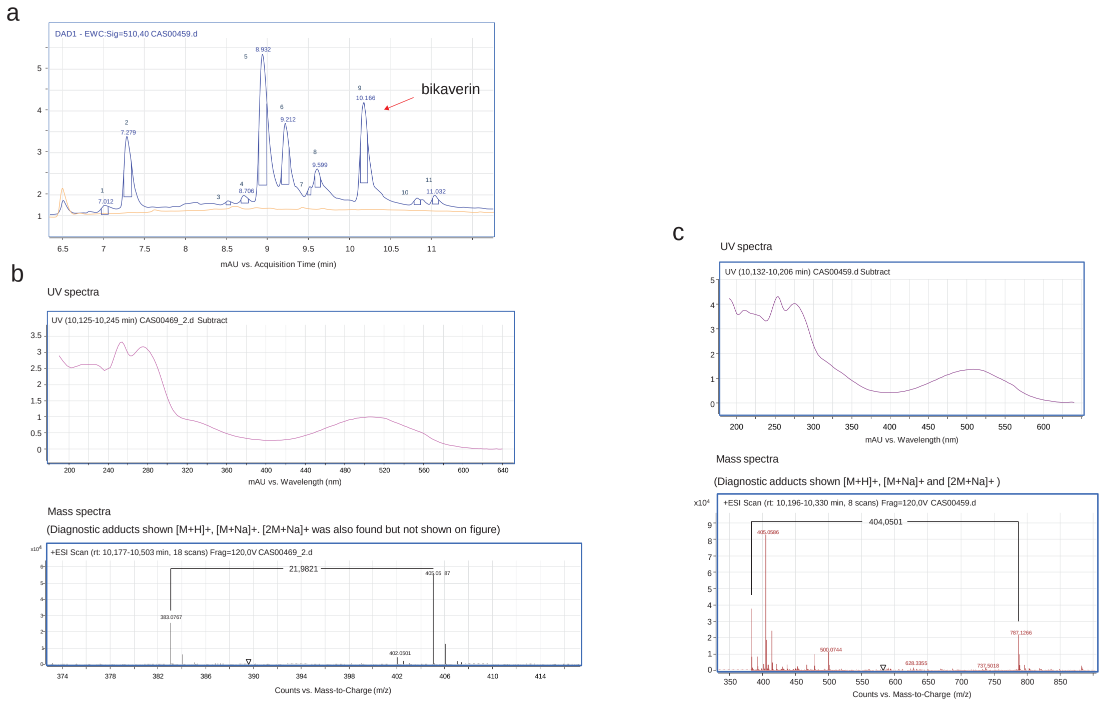

Supplementary Fig. 2. Bikaverin analysis in yZM009 by HPLC, UV spectra and Mass spectra. Strain yZM009 grown in SC–Ura medium with galactose as the carbon source was analyzed by HPLC at 510nm as shown in **a**. (Blue line: yZM009; yellow line: empty control). Red arrow indicted the peak of bikaverin. UV spectra and Mass spectra of bikaverin standard were shown in **b**. The UV spectra and Mass spectra for bikaverin detection in yZM009 were shown in **c**. The UV spectra and Mass spectra were extracted from the peak 9 as shown in **a**.

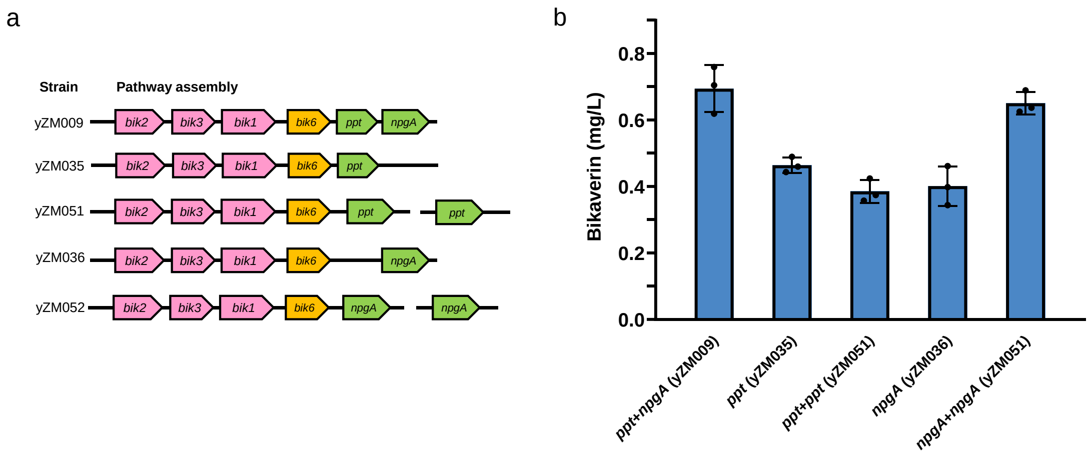

Supplementary Fig. 3. Results from shake-flask fermentation with different strains carrying different copies of Pptase. Genotypes of strains were shown in **a** and titers of bikaverin were shown in **b**. Data are presented as mean values +/– SD and error bars show SD from n=3 biological replicates. Source data underlying Supplementary Figure 3b are provided as a Source Data file.

HPLC/ESI-MS analytical standard

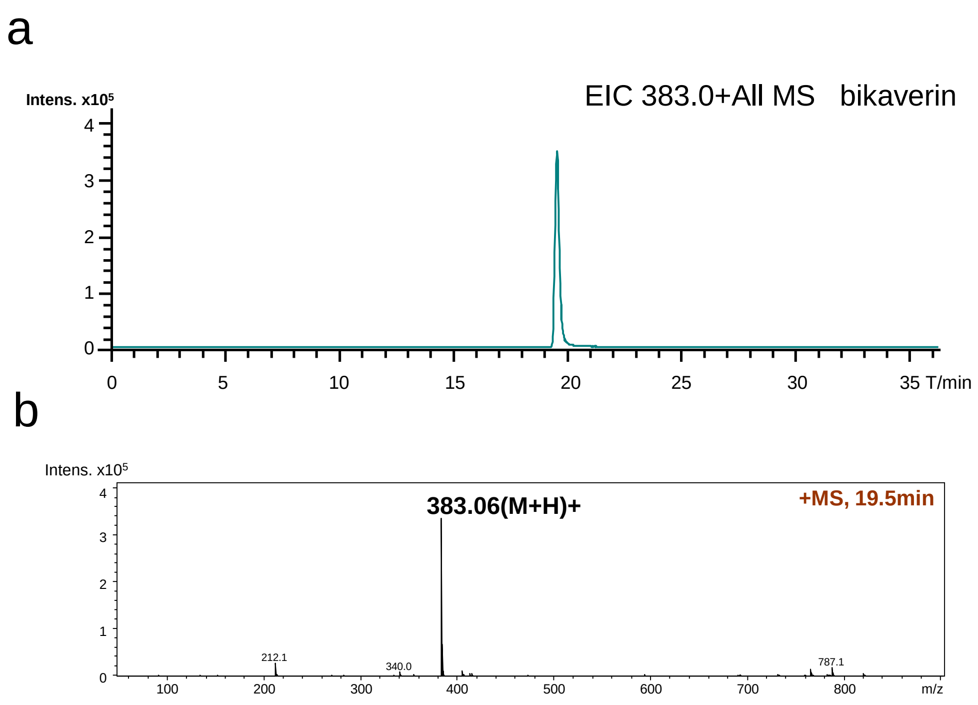

Supplementary Fig. 4. The Bikaverin standard for HPLC/ESI-MS analysis. **a.** the extracted ion chromatogram (EIC) of m/z 383.1**,** the mass of $[M\text{-}H]^+$ of bikaverin. **b.** the mass spectrum of the bikaverin peak in **a**.

EIC 325.0+All MS   Pre-bikaverin

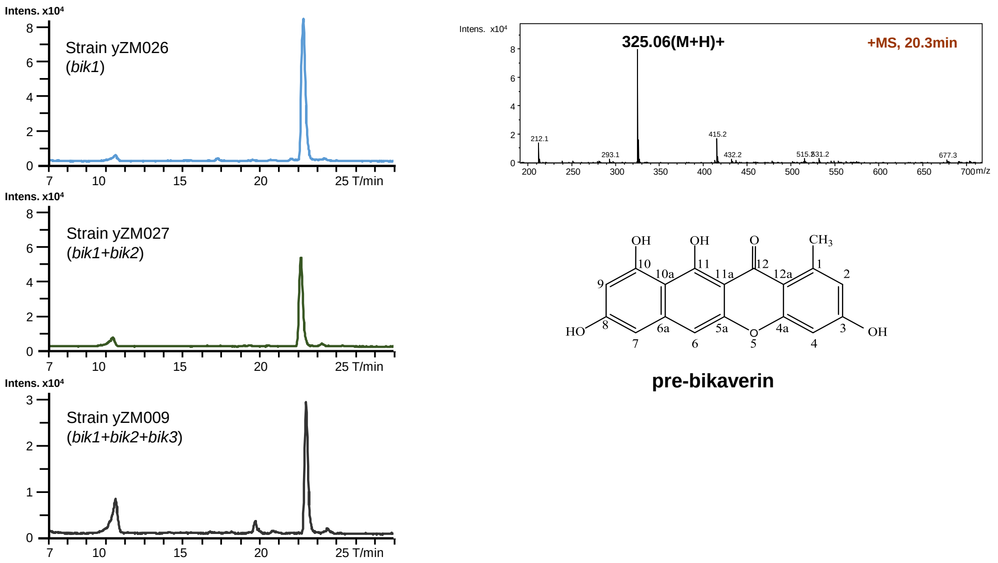

Supplementary Fig. 5. The EIC of pre-bikaverin with m/z 325.1 in strains yZM026, 027 and 009. The mass spectrum of the corresponding peak in yZM026 is shown on the right side.

yZM027-EIC 339.0+All MS   oxo-pre-bikaverin

a

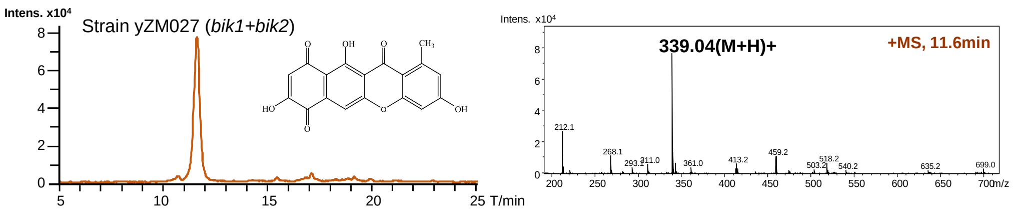

yZM027 EIC 355.0+All MS   Di-nor-bikaverin

b

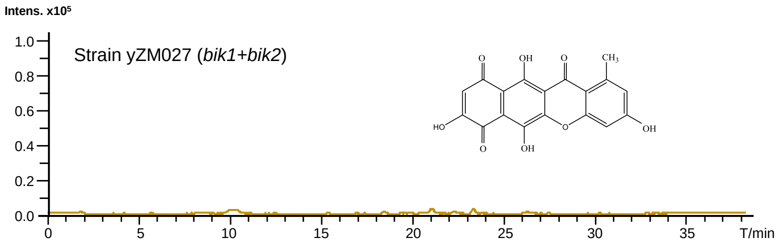

Supplementary Fig. 6. The EIC of oxo-pre-bikaverin and di-nor-bikaverin in strain yZM027. **a.** the peak of oxo-pre-bikaverin, and the corresponding mass spectrum. **b.** the extracted ion chromatogram of m/z 355.0, the mass of $[M\text{-}H]^+$ of di-nor-bikaverin.

yZM028-EIC 339.0+All MS   me-pre-bikaverin

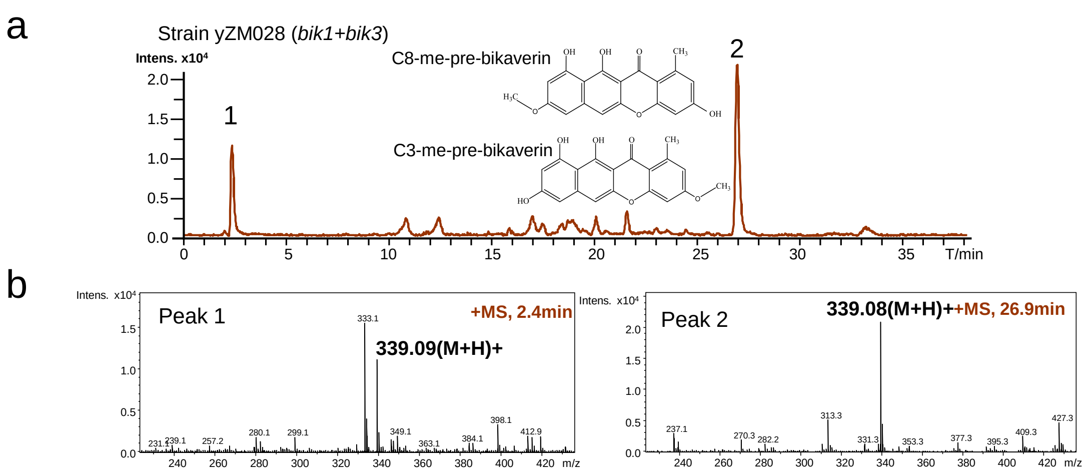

Supplementary Fig. 7. Me-pre-bikaverin detection from strain yZM028. **a.** The two peaks in EIC of me-pre-bikaverin (m/z 339 for $[M\text{-}H]^+$). **b.** The mass spectra of the two peaks.

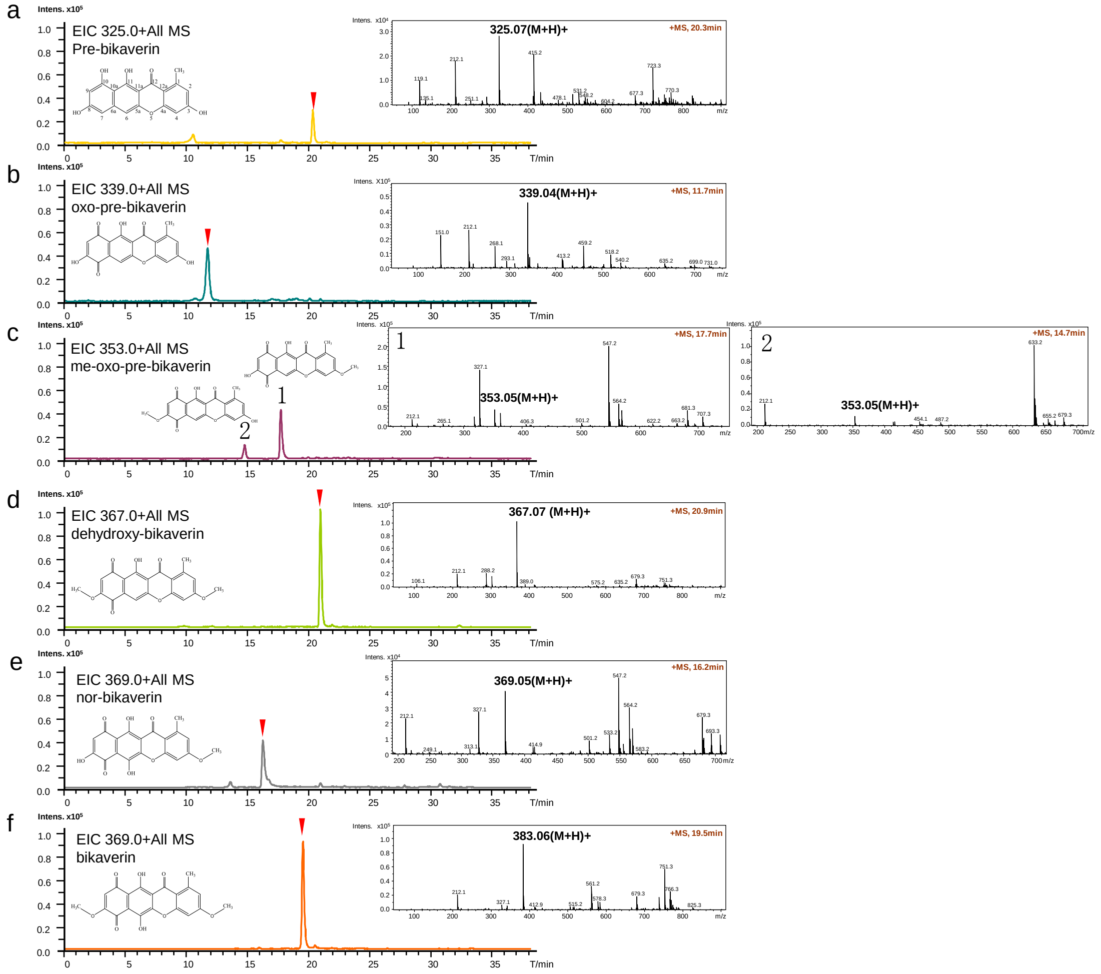

Supplementary Fig. 8. HPLC/ESI-MS spectrum of strain yZM009, with the complete bikaverin pathway. The extracted ion chromatograms and mass spectra of pre-bikaverin, oxo-pre-bikaverin, me-oxo-pre-bikaverin, dehydroxy-bikaverin, nor-bikaverin, bikaverin are shown in **a**, **b**, **c**, **d**, **e** and **f**, respectively.

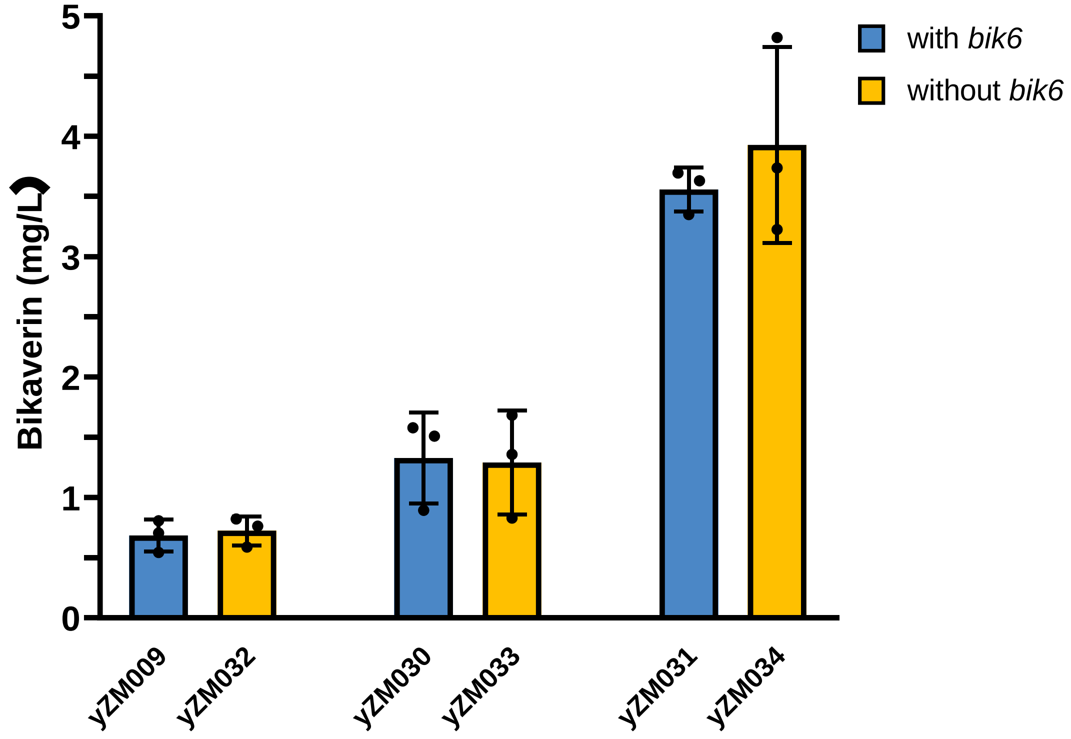

Supplementary Fig. 9. The effect of *bik6* deletion. Strains carrying bikaverin pathways were used to confirm that deletion of *bik6* had little effect on bikaverin production in *S. cerevisiae*. In each group, bikaverin pathways assembled are identical, except for *bik6*. Blue, no *bik6* in bikaverin pathways; yellow, *bik6* was included as the original design. yZM009 and yZM032: P_{GAL1}-*bik1*, P_{TEF1}-*bik2*, P_{TPI1}-*bik3*; yZM030 and yZM033: P_{GAL1}-*bik1*, P_{TEF1}-*bik2*, P_{GAL1}-*bik3*; yZM031 and yZM034: P_{GAL1}-*bik1*, P_{GAL1}-*bik2*, P_{GAL1}-*bik3*. Data are presented as mean values +/– SD and error bars show SD from n=3 biological replicates. Source data are provided as a Source Data file.

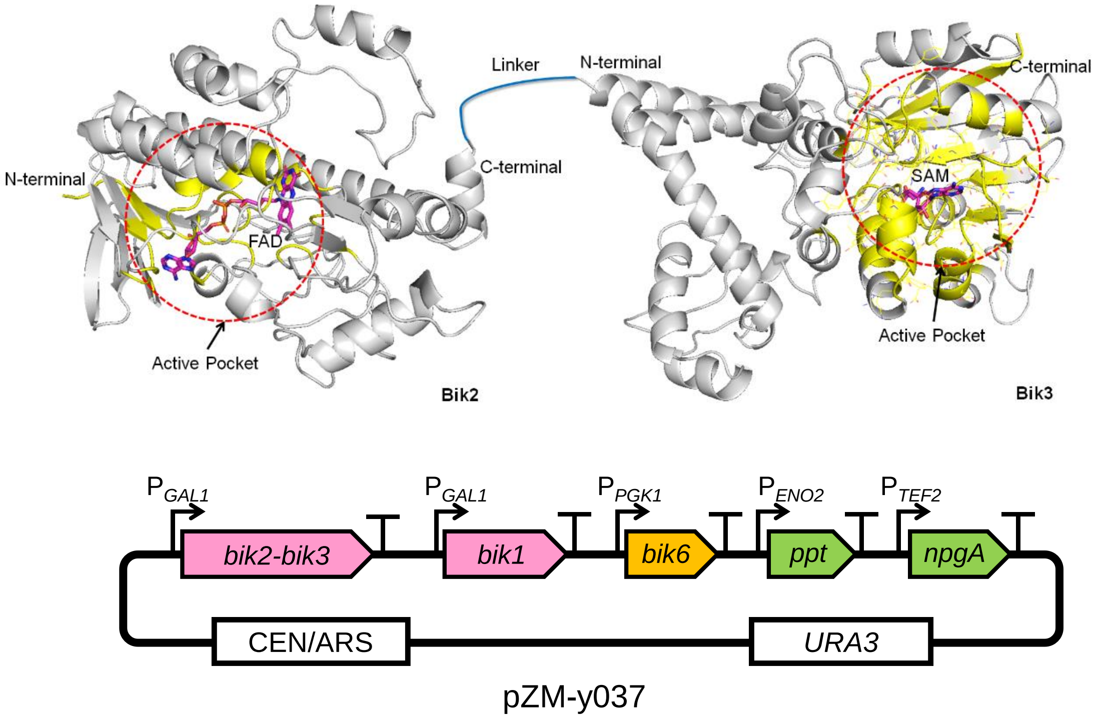

Supplementary Fig. 10. Homology modeling of Bik2 and Bik3. The homology modeling shows that the active pocket and FAD bind site of Bik2 are close to the N-terminal, while for Bik3, its SAM binding site and active pocket are near the C-terminal. In plasmid pZM-y037, proteins were fused in N-Bik2-Bik3-C direction, aiming to minimize the impact on the functions of Bik2 and Bik3.

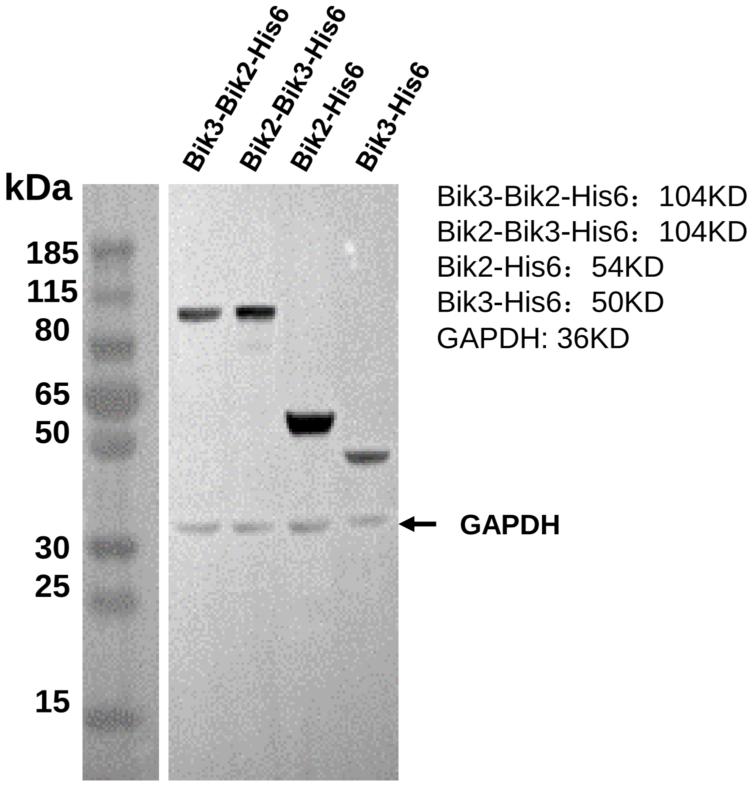

Supplementary Fig. 11. Western blotting of Bik2, Bik3 and fusion proteins. Uncropped scans were supplied in the source data file. All the proteins were tagged with a 6×His-tag and driven by *GAL1* promoter. All strains were cultured in SC–Ura media with galactose as the carbon source. GADPH were used as internal reference protein. Strain yZM044 was used for Bik3-Bik2-His6; strain yZM043 was used for Bik2-Bik3-His6; strain yZM045 was used for Bik2-His6; strain yZM046 was used for Bik3-His6. Experiment was repeated 3 times independently with similar results. Source data are provided as a Source Data file.

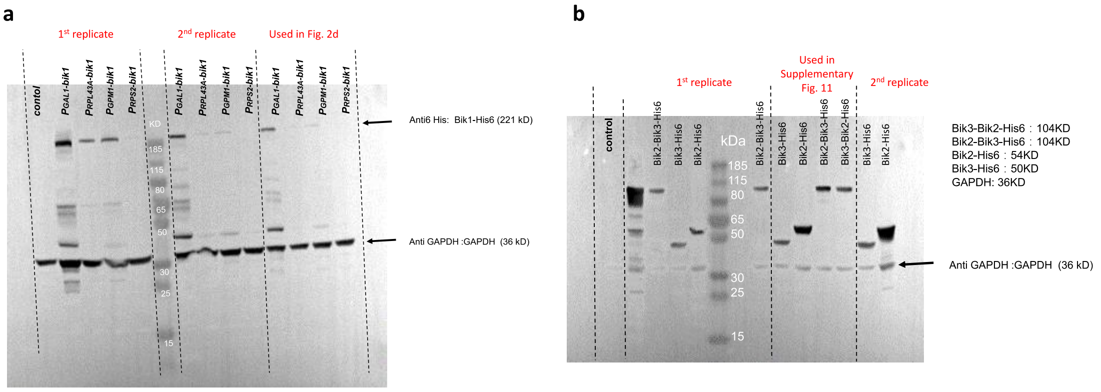

Supplementary Fig. 12. The original uncropped scans of Western blot for Fig. 2 (**a**) and Supplementary Fig. 11 (**b**).

Supplementary Table 1. Yeast strains and coding plasmids for bikaverin pathway expressing in yeast.

Strain	Background	Plasmid	Description	Source
yZM006	BY4742	pZM-y006	pRS416_ P_{TEF1}-BIK2- T_{CYC1}_P_{TPI1}-BIK3- T_{ACS2}_ P_{RPS2}-BIK1- T_{ACS1}_P_{PGK1}-BIK6- T_{ZEO1}_ P_{ENO2}-PPT- T_{ADH2}_P_{TEF2}-NPGA- T_{HXT7}	This study
yZM007	BY4742	pZM-y007	pRS416_ P_{TEF1}-BIK2- T_{CYC1}_P_{TPI1}-BIK3- T_{ACS2}_ P_{RPL43A}-BIK1- T_{ACS1}_P_{PGK1}-BIK6- T_{ZEO1}_ P_{ENO2}-PPT- T_{ADH2}_P_{TEF2}-NPGA- T_{HXT7}	This study
yZM008	BY4742	pZM-y008	pRS416_ P_{TEF1}-BIK2- T_{CYC1}_P_{TPI1}-BIK3- T_{ACS2}_ P_{GPM1}-BIK1- T_{ACS1}_P_{PGK1}-BIK6- T_{ZEO1}_ P_{ENO2}-PPT- T_{ADH2}_P_{TEF2}-NPGA- T_{HXT7}	This study
yZM009	BY4742	pZM-y009	pRS416_ P_{TEF1}-BIK2- T_{CYC1}_P_{TPI1}-BIK3- T_{ACS2}_ P_{GAL1}-BIK1- T_{ACS1}_P_{PGK1}-BIK6- T_{ZEO1}_ P_{ENO2}-PPT- T_{ADH2}_P_{TEF2}-NPGA- T_{HXT7}	This study
yZM010	BY4742	pZM-y010 （Bik1-GFP）	pRS416_ P_{TEF1}-BIK2- T_{CYC1}_P_{TPI1}-BIK3- T_{ACS2}_ P_{RPS2}-BIK1-GFP- T_{ADH1}_ P_{TEF}-KanMX- T_{TEF}- T_{ACS1}_P_{PGK1}-BIK6- T_{ZEO1}_ P_{ENO2}-PPT- T_{ADH2}_P_{TEF2}-NPGA- T_{HXT7} **（derived from pZM006）**	This study
yZM011	BY4742	pZM-y011 （Bik2-GFP）	pRS416_ P_{TEF1}-BIK2-GFP- T_{ADH1}_ P_{TEF}-KanMX- T_{TEF}- T_{CYC1}_P_{TPI1}-BIK3- T_{ACS2}_ P_{RPS2}-BIK1- T_{ACS1}_P_{PGK1}-BIK6- T_{ZEO1}_ P_{ENO2}-PPT- T_{ADH2}_P_{TEF2}-NPGA- T_{HXT7} **（derived from pZM006）**	This study
yZM012	BY4742	pZM-y012 （Bik3-GFP）	pRS416_ P_{TEF1}-BIK2- T_{CYC1}_P_{TPI1}-BIK3-GFP- T_{ADH1}_ P_{TEF}-KanMX- T_{TEF}- T_{ACS2}_ P_{RPS2}-BIK1- T_{ACS1}_P_{PGK1}-BIK6- T_{ZEO1}_ P_{ENO2}-PPT- T_{ADH2}_P_{TEF2}-NPGA- T_{HXT7} **（derived from pZM006）**	This study
yZM013	BY4742	pZM-y013 （Bik6-GFP）	pRS416_ P_{TEF1}-BIK2- T_{CYC1}_P_{TPI1}-BIK3- T_{ACS2}_ P_{RPS2}-BIK1- T_{ACS1}_P_{PGK1}-BIK6-GFP- T_{ADH1}_ P_{TEF}-KanMX- T_{TEF}- T_{ZEO1}_ P_{ENO2}-PPT- T_{ADH2}_P_{TEF2}-NPGA- T_{HXT7} **（derived from pZM006）**	This study
yZM014	BY4742	pZM-y014 （PPT-GFP）	pRS416_ P_{TEF1}-BIK2- T_{CYC1}_P_{TPI1}-BIK3- T_{ACS2}_ P_{RPS2}-BIK1- T_{ACS1}_P_{PGK1}-BIK6- T_{ZEO1}_ P_{ENO2}-PPT-GFP- T_{ADH1}_ P_{TEF}-KanMX- T_{TEF}- T_{ADH2}_P_{TEF2}-NPGA- T_{HXT7} **（derived from pZM006）**	This study
yZM015	BY4742	pZM-y015 （NPGA-GFP）	pRS416_ P_{TEF1}-BIK2- T_{CYC1}_P_{TPI1}-BIK3- T_{ACS2}_ P_{RPS2}-BIK1- T_{ACS1}_P_{PGK1}-BIK6- T_{ZEO1}_ P_{ENO2}-PPT- T_{ADH2}_P_{TEF2}-NPGA- GFP- T_{ADH1}_ P_{TEF}-KanMX- T_{TEF}- T_{HXT7} **（derived from pZM006）**	This study
yZM016	BY4742	pZM-y016 （Bik1-GFP）	pRS416_ P_{TEF1}-BIK2- T_{CYC1}_P_{TPI1}-BIK3- T_{ACS2}_ P_{RPL43A}-BIK1-GFP- T_{ADH1}_ P_{TEF}-KanMX- T_{TEF}- T_{ACS1}_P_{PGK1}-BIK6- T_{ZEO1}_ P_{ENO2}-PPT- T_{ADH2}_P_{TEF2}-NPGA- T_{HXT7} **（derived from pZM007）**	This study
yZM017	BY4742	pZM-y017 （Bik1-GFP）	pRS416_ P_{TEF1}-BIK2- T_{CYC1}_P_{TPI1}-BIK3- T_{ACS2}_ P_{GPM1}-BIK1-GFP- T_{ADH1}_ P_{TEF}-KanMX- T_{TEF}- T_{ACS1}_P_{PGK1}-BIK6- T_{ZEO1}_ P_{ENO2}-PPT- T_{ADH2}_P_{TEF2}-NPGA-	This study

			T_{HXT7} **(derived from pZM008)**	
yZM018	BY4742	pZM-y018 (Bik1-GFP)	pRS416_ P_{TEF1}-BIK2- T_{CYC1}_P_{TPI1}-BIK3- T_{ACS2}_ P_{GAL1}-BIK1- GFP- T_{ADH1}_ P_{TEF}-KanMX- T_{TEF}- T_{ACS1}_P_{PGK1}-BIK6- T_{ZEO1}_ P_{ENO2}-PPT- T_{ADH2}_P_{TEF2}-NPGA- T_{HXT7} **(derived from pZM009)**	This study
yZM021	BY4742	pZM-y006 +pGEV	pRS413-GEV + pRS416_ P_{TEF1}-BIK2- T_{CYC1}_P_{TPI1}-BIK3- T_{ACS2}_ P_{RPS2}-BIK1- T_{ACS1}_P_{PGK1}-BIK6- T_{ZEO1}_ P_{ENO2}-PPT- T_{ADH2}_P_{TEF2}-NPGA- T_{HXT7}	This study
yZM022	BY4742	pZM-y009 +pGEV	pRS413-GEV + pRS416_ P_{TEF1}-BIK2- T_{CYC1}_P_{TPI1}-BIK3- T_{ACS2}_ P_{GAL1}-BIK1- T_{ACS1}_P_{PGK1}-BIK6- T_{ZEO1}_ P_{ENO2}-PPT- T_{ADH2}_P_{TEF2}-NPGA- T_{HXT7}	This study
yZM026	BY4742	pZM-y026	pRS416_ P_{GAL1}-BIK1-T_{ACS1}_P_{PGK1}-BIK6- T_{ZEO1}_ P_{ENO2}-PPT- T_{ADH2}_P_{TEF2}-NPGA- T_{HXT7}	This study
yZM027	BY4742	pZM-y027	pRS416_ P_{TEF1}-BIK2- T_{CYC1} _ P_{GAL1}-BIK1-T_{ACS1}_P_{PGK1}-BIK6- T_{ZEO1}_ P_{ENO2}-PPT- T_{ADH2}_P_{TEF2}-NPGA- T_{HXT7}	This study
yZM028	BY4742	pZM-y028	pRS416_ P_{GAL1}-BIK3- T_{ACS2}_ P_{GAL1}-BIK1-T_{ACS1}_P_{PGK1}-BIK6- T_{ZEO1}_ P_{ENO2}-PPT- T_{ADH2}_P_{TEF2}-NPGA- T_{HXT7}	This study
yZM029	BY4742	pZM-y029	pRS416_ P_{GAL1}-BIK2- T_{CYC1}_P_{TPI1}-BIK3- T_{ACS2}_ P_{GAL1}-BIK1- T_{ACS1}_P_{PGK1}-BIK6- T_{ZEO1}_ P_{ENO2}-PPT- T_{ADH2}_P_{TEF2}-NPGA- T_{HXT7}	This study
yZM030	BY4742	pZM-y030	pRS416_ P_{TEF1}-BIK2- T_{CYC1}_P_{GAL1}-BIK3- T_{ACS2}_ P_{GAL1}-BIK1-T_{ACS1}_P_{PGK1}-BIK6- T_{ZEO1}_ P_{ENO2}-PPT- T_{ADH2}_P_{TEF2}-NPGA- T_{HXT7}	This study
yZM031	BY4742	pZM-y031	pRS416_ P_{GAL1}-BIK2- T_{CYC1}_P_{GAL1}-BIK3- T_{ACS2}_ P_{GAL1}-BIK1-T_{ACS1}_P_{PGK1}-BIK6- T_{ZEO1}_ P_{ENO2}-PPT- T_{ADH2}_P_{TEF2}-NPGA- T_{HXT7}	This study
yZM032	BY4742	pZM-y032	pRS416_ P_{TEF1}-BIK2- T_{CYC1}_P_{TPI1}-BIK3- T_{ACS2}_ P_{GAL1}-BIK1-T_{ACS1}_ P_{ENO2}-PPT- T_{ADH2}_P_{TEF2}-NPGA- T_{HXT7}	This study
yZM033	BY4742	pZM-y033	pRS416_ P_{TEF1}-BIK2- T_{CYC1}_P_{GAL1}-BIK3- T_{ACS2}_ P_{GAL1}-BIK1-T_{ACS1}_ P_{ENO2}-PPT- T_{ADH2}_P_{TEF2}-NPGA- T_{HXT7}	This study
yZM034	BY4742	pZM-y034	pRS416_ P_{GAL1}-BIK2- T_{CYC1}_P_{GAL1}-BIK3- T_{ACS2}_ P_{GAL1}-BIK1-T_{ACS1}_ P_{ENO2}-PPT- T_{ADH2}_P_{TEF2}-NPGA- T_{HXT7}	This study
yZM035	BY4742	pZM-y035	pRS416_ P_{TEF1}-BIK2- T_{CYC1}_P_{TPI1}-BIK3- T_{ACS2}_ P_{GAL1}-BIK1- T_{ACS1}_P_{PGK1}-BIK6- T_{ZEO1}_ P_{ENO2}-PPT- T_{ADH2}	This study
yZM036	BY4742	pZM-y036	pRS416_ P_{TEF1}-BIK2- T_{CYC1}_P_{TPI1}-BIK3- T_{ACS2}_ P_{GAL1}-BIK1- T_{ACS1}_P_{PGK1}-BIK6- T_{ZEO1}_ P_{ENO2}-NPGA- T_{ADH2}	This study
yZM037	BY4742	pZM-y037	pRS416_ P_{GAL1}-BIK2-BIK3- T_{CYC1}_ P_{GAL1}-BIK1-T_{ACS1}_ P_{PGK1}-BIK6- T_{ZEO1}_P_{ENO2}-PPT- T_{ADH2}_P_{TEF2}-NPGA- T_{HXT7}	This study
yZM038	BY4742	pZM-y038	pRS416_ P_{GAL1}-BIK2-BIK3-GFP- T_{ADH1}_ P_{TEF}-KanMX- T_{TEF}- T_{CYC1}_ P_{GAL1}-BIK1-T_{ACS1}_ P_{PGK1}-BIK6- T_{ZEO1}_P_{ENO2}-PPT- T_{ADH2}_P_{TEF2}-NPGA- T_{HXT7} **(derived from pZM037)**	This study
yZM039	BY4742	pZM-y039	pRS416_ P_{GAL1}-BIK2-GFP- T_{ADH1}_ P_{TEF}-KanMX- T_{TEF} - T_{CYC1}_P_{GAL1}-BIK3- T_{ACS2}_ P_{GAL1}-BIK1- GFP-T_{ACS1}_P_{PGK1}-BIK6- T_{ZEO1}_ P_{ENO2}-PPT- T_{ADH2}_P_{TEF2}-NPGA- T_{HXT7} **(derived from pZM031)**	This study
yZM040	BY4742	pZM-y040	pRS416_ P_{GAL1}-BIK2- T_{CYC1}_P_{GAL1}-BIK3-GFP- T_{ADH1}_ P_{TEF}-KanMX- T_{TEF} - T_{ACS2}_ P_{GAL1}-BIK1- GFP-T_{ACS1}_P_{PGK1}-BIK6- T_{ZEO1}_ P_{ENO2}-PPT-	This study

			T_{ADH2}_P_{TEF2}-NPGA- T_{HXT7} **(derived from pZM031)**	
yZM041	BY4742	pZM-y041	pRS416_ P_{TEF1}-BIK2- T_{CYC1}_P_{TPI1}-BIK3- T_{ACS2}_ P_{GAL1}-BIK1- T_{ACS1}_P_{PGK1}-BIK6- T_{ZEO1}	This study
yZM042	BY4742	pZM-y042	pRS416_ P_{GAL1}-BIK3-BIK2- T_{CYC1}_ P_{GAL1}-BIK1-T_{ACS1}_ P_{PGK1}-BIK6- T_{ZEO1}_P_{ENO2}-PPT- T_{ADH2}_P_{TEF2}-NPGA- T_{HXT7}	This study
yZM043	BY4742		pRS416_ P_{GAL1}-BIK2-BIK3-6xHis-tag- T_{ADH1}_ P_{TEF}-KanMX- T_{TEF}- T_{CYC1}_ P_{GAL1}-BIK1-T_{ACS1}_ P_{PGK1}-BIK6- T_{ZEO1}_P_{ENO2}-PPT- T_{ADH2}_P_{TEF2}-NPGA- T_{HXT7} **(derived from pZM037)**	This study
yZM044	BY4742		pRS416_ P_{GAL1}-BIK3-BIK2-6xHis-tag - T_{ADH1}_ P_{TEF}-KanMX- T_{TEF}- T_{CYC1}_ P_{GAL1}-BIK1-T_{ACS1}_ P_{PGK1}-BIK6- T_{ZEO1}_P_{ENO2}-PPT- T_{ADH2}_P_{TEF2}-NPGA- T_{HXT7} **(derived from pZM042)**	This study
yZM045	BY4742		pRS416_ P_{GAL1}-BIK2-6xHis-tag - T_{ADH1}_ P_{TEF}-KanMX- T_{TEF} - T_{CYC1}_P_{GAL1}-BIK3- T_{ACS2}_ P_{GAL1}-BIK1- GFP-T_{ACS1}_P_{PGK1}-BIK6- T_{ZEO1}_ P_{ENO2}-PPT- T_{ADH2}_P_{TEF2}-NPGA- T_{HXT7} **(derived from pZM031)**	This study
yZM046	BY4742		pRS416_ P_{GAL1}-BIK2- T_{CYC1}_P_{GAL1}-BIK3-6xHis-tag - T_{ADH1}_ P_{TEF}-KanMX- T_{TEF} - T_{ACS2}_ P_{GAL1}-BIK1- GFP-T_{ACS1}_P_{PGK1}-BIK6- T_{ZEO1}_ P_{ENO2}-PPT- T_{ADH2}_P_{TEF2}-NPGA- T_{HXT7} **(derived from pZM031)**	This study
yZM047	BY4742		pRS416_ P_{TEF1}-BIK2- T_{CYC1}_P_{TPI1}-BIK3- T_{ACS2}_ P_{RPS2}-BIK1- 6xHis-tag - T_{ADH1}_ P_{TEF}-KanMX- T_{TEF} - T_{ACS1}_P_{PGK1}-BIK6- T_{ZEO1}_ P_{ENO2}-PPT- T_{ADH2}_P_{TEF2}-NPGA- T_{HXT7} **(derived from pZM006)**	This study
yZM048	BY4742		pRS416_ P_{TEF1}-BIK2- T_{CYC1}_P_{TPI1}-BIK3- T_{ACS2}_ P_{GPM1}-BIK1- 6xHis-tag - T_{ADH1}_ P_{TEF}-KanMX- T_{TEF} - T_{ACS1}_P_{PGK1}-BIK6- T_{ZEO1}_ P_{ENO2}-PPT- T_{ADH2}_P_{TEF2}-NPGA- T_{HXT7} **(derived from pZM008)**	This study
yZM049	BY4742		pRS416_ P_{TEF1}-BIK2- T_{CYC1}_P_{TPI1}-BIK3- T_{ACS2}_ P_{RPL43A}-BIK1- 6xHis-tag - T_{ADH1}_ P_{TEF}-KanMX- T_{TEF} - T_{ACS1}_P_{PGK1}-BIK6- T_{ZEO1}_ P_{ENO2}-PPT- T_{ADH2}_P_{TEF2}-NPGA- T_{HXT7} **(derived from pZM007)**	This study
yZM050	BY4742		pRS416_ P_{TEF1}-BIK2- T_{CYC1}_P_{TPI1}-BIK3- T_{ACS2}_ P_{GAL1}-BIK1-6xHis-tag - T_{ADH1}_ P_{TEF}-KanMX- T_{TEF} - T_{ACS1}_P_{PGK1}-BIK6- T_{ZEO1}_ P_{ENO2}-PPT- T_{ADH2}_P_{TEF2}-NPGA- T_{HXT7} **(derived from pZM009)**	This study
yZM051	BY4742	pZM035+bZM027		This study
yZM052	BY4742	pZM036+bZM028		This study

Supplementary Table 2. *E. coli* strains and plasmids used in this study.

Plasmid	Description	Source
bZM007	*pUC19-VA1-*P_{TEF1}*-BIK2-* T_{CYC1}*-VA3*	This study
bZM008	*pUC19-VA3-* P_{TPI1}*-BIK3-* T_{ACS2}*-VA4*	This study
bZM009	*pUC19-VA4-* P_{RPS2}*-BIK1-* T_{ACS1}*-VA5*	This study
bZM010	*pUC19-VA5-* P_{PGK1}*-BIK6-* T_{ZEO1}*-VA6*	This study
bZM011	*pUC19-VA6-* P_{ENO2}*-PPT-* T_{ADH2}*-VA7*	This study
bZM012	*pUC19-VA7-* P_{TEF2}*-NPGA-* T_{HXT7}*-VA2*	This study
bZM013	*pUC19-VA6-* P_{ENO2}*-PPT-* T_{ADH2}*-VA2*	This study
bZM014	*pUC19-VA6-* P_{ENO2}*-NPGA-* T_{ADH2}*-VA2*	This study
bZM015	*pUC19-VA5-* P_{ENO2}*-PPT-* T_{ADH2}*-VA7*	This study
bZM016	*pUC19-VA5-* P_{ENO2}*-PPT-* T_{ADH2}*-VA7*	This study
bZM017	*pRS416-VA3-VA4- RFP-VA2*	This study
pJC170	*pRS416-VA1-RFP-VA2*	Mitchell *et al.*[1]
bZM019	*pUC19-VA1-*P_{GAL1}*-BIK2-* T_{CYC1}*-VA3*	This study
bZM020	*pUC19-VA3-* P_{GAL1}*-BIK3-* T_{ACS2}*-VA4*	This study
bZM021	*pUC19-VA4-* P_{GAL1}*-BIK1-* T_{ACS1}*-VA5*	This study
bZM023	*pUC19-VA4-* P_{RPL43A}*-BIK1-* T_{ACS1}*-VA5*	This study
bZM024	*pUC19-VA4-* P_{GPM1}*-BIK1-* T_{ACS1}*-VA5*	This study
bZM026	*pUC19-VA1-*P_{GAL1}*-BIK2-BIK3 -*T_{CYC1}*-VA3*	This study
bZM027	*pRS413- VA6-* P_{ENO2}*-PPT-* T_{ADH2}*-VA2*	This study
bZM028	*pRS413- VA6-* P_{ENO2}*-NPGA-* T_{ADH2}*-VA2*	This study
pGEV	*pGal4-ER-VP16-HIS3*	Cai *et al.*[2;] McIsaac *et al.* [3]

Supplementary Table 3. Primer sequences used in this study.

Primer	Sequence
Bik2-GFP-F	CCAGTTCAAGCTGCTACCGGTGTTGTTGAAGTTGGTTCTGGTCGACGGATCCCCGGGTT
Bik2-GFP-R	AACTAATTACATGATATCGACAAAGGAAAAGGGGCCTGTTCGATGAATTCGAGCTCGTT
Bik3-GFP-F	TACGGTTCTTTCATGTCTGTTATCGACGTTGTTTTGGGTGGTCGACGGATCCCCGGGTT
Bik3-GFP-R	CGAAATTTTATCTCATTACGAAATTTTTCTCATTTAAGTTCGATGAATTCGAGCTCGTT
Bik1-GFP-F	GCTTTGTGTGCTAAGATCAGAGAAACCATGGGTGTTAACGGTCGACGGATCCCCGGGTT
Bik1-GFP-R	AAAAAAAAGTCGTCAATATAAAAAGGAAAGAAATCATCATCGATGAATTCGAGCTCGTT
Bik6-GFP-F	ATCAGAGCTAGAGGTGAATTCTCTAAGTTGTCTACCTACGGTCGACGGATCCCCGGGTT
Bik6-GFP-R	AAAGAAACTTCTAGTAAAGTGCAGCACATTCAAGTGTGATCGATGAATTCGAGCTCGTT
PPT-GFP-F	GAAGAAATCTTGGCTTTCGGTGAACAAGCTTCTAAGCCAGGTCGACGGATCCCCGGGTT
PPT-GFP-R	AATGAAAACTATAAATCGTAAAGACATAAGAGATCCGCTTCGATGAATTCGAGCTCGTT
NPGA-GFP-F	ATCCAACCATGTGCTACCGGTGTTTGTAACTGTTTGTCTGGTCGACGGATCCCCGGGTT
NPGA-GFP-R	ATTAGAGCGTGATCATGAATTAATAAAAGTGTTCGCAAATCGATGAATTCGAGCTCGTT
His-tag-F	CACCACCATCACCATCACTAAGCGAATTTCTTATGATTTATGATTTTTATTAT
His-tag-R	TCGATGAATTCGAGCTCGTT
Bik2-6xhis-F	CCAGTTCAAGCTGCTACCGGTGTTGTTGAAGTTGGTTCTCACCACCATCACCATCAC TAAGC
Bik2-6xhis-R	AACTAATTACATGATATCGACAAAGGAAAAGGGGCCTGTTCGATGAATTCGAGCTCGTT
Bik3-6xhis-F	TACGGTTCTTTCATGTCTGTTATCGACGTTGTTTTGGGTCACCACCATCACCATCAC TAAGC
Bik3-6xhis-R	CGAAATTTTATCTCATTACGAAATTTTTCTCATTTAAGTTCGATGAATTCGAGCTCGTT
Bik1-6xhis-F	GCTTTGTGTGCTAAGATCAGAGAAACCATGGGTGTTAACCACCACCATCACCATCAC TAAGC
Bik1-6xhis-R	AAAAAAAAGTCGTCAATATAAAAAGGAAAGAAATCATCATCGATGAATTCGAGCTCGTT
Bik6-6xhis-F	ATCAGAGCTAGAGGTGAATTCTCTAAGTTGTCTACCTACCACCACCATCACCATCAC TAAGC
Bik6-6xhis-R	AAAGAAACTTCTAGTAAAGTGCAGCACATTCAAGTGTGATCGATGAATTCGAGCTCGTT
PPT-6xhis-F	GAAGAAATCTTGGCTTTCGGTGAACAAGCTTCTAAGCCACACCACCATCACCATCAC TAAGC
PPT-6xhis-R	AATGAAAACTATAAATCGTAAAGACATAAGAGATCCGCTTCGATGAATTCGAGCTCGTT
NPGA-6xhis-F	ATCCAACCATGTGCTACCGGTGTTTGTAACTGTTTGTCTCACCACCATCACCATCAC TAAGC
NPGA-6xhis-R	ATTAGAGCGTGATCATGAATTAATAAAAGTGTTCGCAAATCGATGAATTCGAGCTCGTT
Bik2-6xhis-F	CCAGTTCAAGCTGCTACCGGTGTTGTTGAAGTTGGTTCTCACCACCATCACCATCAC TAAGC
Bik2-6xhis-R	AACTAATTACATGATATCGACAAAGGAAAAGGGGCCTGTTCGATGAATTCGAGCTCGTT
Bik3-6xhis-F	TACGGTTCTTTCATGTCTGTTATCGACGTTGTTTTGGGTCACCACCATCACCATCAC TAAGC
Bik3-6xhis-R	CGAAATTTTATCTCATTACGAAATTTTTCTCATTTAAGTTCGATGAATTCGAGCTCGTT
Bik1-6xhis-F	GCTTTGTGTGCTAAGATCAGAGAAACCATGGGTGTTAACCACCACCATCACCATCAC TAAGC
Bik1-6xhis-R	AAAAAAAAGTCGTCAATATAAAAAGGAAAGAAATCATCATCGATGAATTCGAGCTCGTT

Note: The sequences underlined represent the homology arms used to promote yeast homologous recombination.

Supplementary References

1. Mitchell LA, *et al.* Versatile genetic assembly system (VEGAS) to assemble pathways for expression in *S. cerevisiae*. *Nucleic Acids Res* **43**, 6620-6630 (2015).
2. Cai Y, *et al.* Intrinsic biocontainment: multiplex genome safeguards combine transcriptional and recombinational control of essential yeast genes. *Proc Natl Acad Sci U S A* **112**, 1803-1808 (2015).
3. McIsaac RS, *et al.* Fast-acting and nearly gratuitous induction of gene expression and protein depletion in *Saccharomyces cerevisiae*. *Mol Biol Cell* **22**, 4447-4459 (2011).
