# Supplementary material for: Pathway engineering in yeast for synthesizing the complex polyketide bikaverin
Source: Nat Commun. 2020 Dec 3;11:6197. doi: 10.1038/s41467-020-19984-3 (PMC7713123; doi:10.1038/s41467-020-19984-3)
Supplement: Supplementary file 3 — Description of Additional Supplementary Files [file 41467_2020_19984_MOESM3_ESM.pdf]

Description of Additional Supplementary Files

File name: Supplementary Data 1

Description: the DNA sequences used in bikaverin pathway
