# Supplementary material for: Pathway engineering in yeast for synthesizing the complex polyketide bikaverin
Source: Nat Commun. 2020 Dec 3;11:6197. doi: 10.1038/s41467-020-19984-3 (PMC7713123; doi:10.1038/s41467-020-19984-3)
Supplement: Supplementary file 5 — Reporting Summary [file 41467_2020_19984_MOESM5_ESM.pdf]

nature research

Corresponding author(s): Jef D. Boeke

Last updated by author(s): Sep 18, 2020

Reporting Summary

Nature Research wishes to improve the reproducibility of the work that we publish. This form provides structure for consistency and transparency in reporting. For further information on Nature Research policies, see our Editorial Policies and the Editorial Policy Checklist.

Statistics

For all statistical analyses, confirm that the following items are present in the figure legend, table legend, main text, or Methods section.

n/a	Confirmed	
☐	☒	The exact sample size (*n*) for each experimental group/condition, given as a discrete number and unit of measurement
☐	☒	A statement on whether measurements were taken from distinct samples or whether the same sample was measured repeatedly
☒	☐	The statistical test(s) used AND whether they are one- or two-sided<br>*Only common tests should be described solely by name; describe more complex techniques in the Methods section.*
☒	☐	A description of all covariates tested
☒	☐	A description of any assumptions or corrections, such as tests of normality and adjustment for multiple comparisons
☐	☒	A full description of the statistical parameters including central tendency (e.g. means) or other basic estimates (e.g. regression coefficient) AND variation (e.g. standard deviation) or associated estimates of uncertainty (e.g. confidence intervals)
☒	☐	For null hypothesis testing, the test statistic (e.g. *F*, *t*, *r*) with confidence intervals, effect sizes, degrees of freedom and *P* value noted<br>*Give P values as exact values whenever suitable.*
☒	☐	For Bayesian analysis, information on the choice of priors and Markov chain Monte Carlo settings
☒	☐	For hierarchical and complex designs, identification of the appropriate level for tests and full reporting of outcomes
☒	☐	Estimates of effect sizes (e.g. Cohen's *d*, Pearson's *r*), indicating how they were calculated

Our web collection on statistics for biologists contains articles on many of the points above.

Software and code

Policy information about availability of computer code

Data collection

No custom software was used. Data collection is aslo described in Methods section.
Green fluorescence pictures (Fig. 2b, c) were collected using EVOS-FL Auto cell imaging system (Invitrogen) with the 20X objective lens. HPLC detection of bikaverin was performed on a Water 2695 system equipped with a 2489 UV detector operating at 510 nm. For ESI-MS data, Bruker micrOTOF-Q II instrument connected with Agilent HPLC system (Agilent G1312B SL binary; Agilent G1367C SL WP) were used to detect bikaverin and pathway intermediates. Western blots pictures were collected by Tanon-4800 Muti imaging system. Protein sequences were reverse-translated into nucleotide sequences and codon optimized for expression in yeast using online tool BioPartsBuilder (http://public.biopartsbuilder.org, no version informations).

Data analysis

Data analysis is described in the Methods section. HPLC data was analyzed with Water Empower 3.0 Chromatography Data Software. The Mass data was analyzed with Bruker Compass Data Analysis software (version 4.0) .To explore the effect of protein fusion version of Bik2 and Bik3, the tridimensional structure models of Bik2, Bik3 and forward fusion-protein Bik2-Bik3 were built and optimized by EasyModeller (Version 4.0). The high-resolution complex structures of rifampicin monooxygenase with FAD (PBDID: 5KOW) and methyltransferase with SAM (PBDID: 5w7p-A) were used as the templates of Bik2 and Bik3, respectively. Modeled structures were analyzed using Pymol software (Version 1.7).The Western blots results were quantified using ImageJ (Version 1.52a) software (https://imagej.nih.gov/ij/).

For manuscripts utilizing custom algorithms or software that are central to the research but not yet described in published literature, software must be made available to editors and reviewers. We strongly encourage code deposition in a community repository (e.g. GitHub). See the Nature Research guidelines for submitting code & software for further information.

Data

Policy information about availability of data

All manuscripts must include a data availability statement. This statement should provide the following information, where applicable:

- Accession codes, unique identifiers, or web links for publicly available datasets
- A list of figures that have associated raw data
- A description of any restrictions on data availability

The protein sequences of Bik1, Bik2, Bik3, Bik6, Ppt1 and NpgA were obtained from the NCBI Protein database (accession numbers: Bik1, S0DZM7; Bik2, S0E2X6; Bik3, S0E608; Bik6, S0DZN4; Ppt1, CCE73639; NpgA, AAF12814). Their DNA sequences used in this study were provided in supplementary data 1. The templates of Bik2 and Bik3 used for homology modeling were obtained from RCBS-PDB database (PDB ID: 5KOW and 5w7p-A, respectively). The LCMS spectra data are available in Supplementary Figure 4-8. Other data and materials used in this study are available upon request.

Field-specific reporting

Please select the one below that is the best fit for your research. If you are not sure, read the appropriate sections before making your selection.

- [x] Life sciences
- [ ] Behavioural & social sciences
- [ ] Ecological, evolutionary & environmental sciences

For a reference copy of the document with all sections, see nature.com/documents/nr-reporting-summary-flat.pdf

Life sciences study design

All studies must disclose on these points even when the disclosure is negative.

Sample size	No statistical methods were used to predetermined sample size. A minimum of N = 3 biological replicates were tested to provide sufficient reproducibility.
Data exclusions	All data are included for analysis.
Replication	All assays including fluorescence microscopy, western blot, HPLC, fermentation and bikaverin titer determination were repeated 2-4 times independently and showed similar results.
Randomization	To measure the bikaverin yield, three single colonies transformed with the bikaverin synthesis pathway or empty vector were randomly picked up as biological replicates. The sample allocation was not available because patient or human population data was not involved in this study.
Blinding	Blinding is not relevant because no group allocation was involved in this study.

Reporting for specific materials, systems and methods

We require information from authors about some types of materials, experimental systems and methods used in many studies. Here, indicate whether each material, system or method listed is relevant to your study. If you are not sure if a list item applies to your research, read the appropriate section before selecting a response.

Materials & experimental systems

n/a	Involved in the study	
[ ]	[x]	Antibodies
[x]	[ ]	Eukaryotic cell lines
[x]	[ ]	Palaeontology and archaeology
[x]	[ ]	Animals and other organisms
[x]	[ ]	Human research participants
[x]	[ ]	Clinical data
[x]	[ ]	Dual use research of concern

Methods

n/a	Involved in the study	
[x]	[ ]	ChIP-seq
[x]	[ ]	Flow cytometry
[x]	[ ]	MRI-based neuroimaging

Antibodies

Antibodies used	ProteinFind® Anti-His Mouse Monoclonal Antibody(HT501,TransGen Biotech, China);<br>ProteinFind® Anti-GAPDH Mouse Monoclonal Antibody(HC301,TransGen Biotech, China);<br>ProteinFind® Goat Anti-Mouse IgG (H+L), HRP Conjugate(HS201,TransGen Biotech, China)
Validation	All antibodies are commercially available and commonly used. These commercially available antibodies were all validated by the producers.<br><br>ProteinFind® Anti-His Mouse Monoclonal Antibody:

Anti-his label mouse monoclonal antibody is a high purity mouse monoclonal antibody, belonging to IgG1 homotype, immunized with a synthetic 6×His label polypeptide sequence (HHHHHH).This antibody is highly specific for the identification of 6×His tags at the C-terminal or N-terminal of recombinant proteins and is suitable for the qualitative or quantitative detection of His fusion expressed proteins.   Species Reactivity: Bacteria, Yeast   Applications:WB, ELISA, IF,IP.

This antibody was validated by the producers and had been used in following references:

Zhang B , Yang Q , Chen J , et al. CRISPRi-Manipulation of Genetic Code Expansion via RF1 for Reassignment of Amber Codon in Bacteria[J]. Scientific Reports, 2016, 6:20000.

Li ST, Wang N, Xu S , et al. Quantitative study of yeast Alg1 beta-1, 4 mannosyltransferase activity, a key enzyme involved in protein N- glycosylation[J]. BBA - General Subjects, 2017, 1861(1):2934-2941.

Nan Liang, Chen Chen, Ying Wang, et al. Exploring Catalysis Specificity of Phytoene Dehydrogenase CrtI in Carotenoid Synthesis ACS Synthetic Biology 2020 9 (7):1753-1762

PProteinFind® Anti-GAPDH Mouse Monoclonal Antibody:

Anti-gapdh mouse monoclonal antibody is a high-purity anti-mouse monoclonal antibody. It belongs to IgG1 homotype and is immunized with full-length HUMAN GAPDH protein.This antibody can recognize GAPDH protein from human, rat, mouse, rabbit and other species.GAPDH protein derived from species such as rabbits. Applications:WB, ELISA, IF,IP.

This antibody was validated by the producers and had been used in following references:

References: Zhang T , Lin Y , Liu J , et al. Rbm24 Regulates Alternative Splicing Switch in Embryonic Stem Cell Cardiac Lineage Differentiation[J]. STEM CELLS, 2016, 34(7):1776-1789.

Li Q , Li B , Hu L , et al. Identification of a novel functional JAK1 S646P mutation in acute lymphoblastic leukemia[J]. Oncotarget, 2017, 8(21):34687-34697.

Nan Liang, Chen Chen, Ying Wang, et al. Exploring Catalysis Specificity of Phytoene Dehydrogenase CrtI in Carotenoid Synthesis ACS Synthetic Biology 2020 9 (7):1753-1762

ProteinFind® Goat Anti-Mouse IgG (H+L), HRP Conjugate:

Affinity purified ProteinFind® Goat Anti-Mouse IgG(H+L) Antibody is a horseradish peroxidase (HRP) conjugated secondary antibody for ELISA and Western blot detection.   Applications:WB, ELISA,

This antibody was validated by the producers and had been used in following references:

Fan L , Cao X , Yan H , et al. The synthetic antihyperlipidemic drug potassium piperate selectively kills breast cancer cells through inhibiting G1-S-phase transition and inducing apoptosis[J]. Oncotarget, 2017, 8(29):47250-47268.

Nan Liang, Chen Chen, Ying Wang, et al. Exploring Catalysis Specificity of Phytoene Dehydrogenase CrtI in Carotenoid Synthesis ACS Synthetic Biology 2020 9 (7):1753-1762
