# Supplementary material for: Pathway engineering in yeast for synthesizing the complex polyketide bikaverin
Source: Nat Commun. 2020 Dec 3;11:6197. doi: 10.1038/s41467-020-19984-3 (PMC7713123; doi:10.1038/s41467-020-19984-3)
Supplement: Supplementary file 2 — Peer Review File [file 41467_2020_19984_MOESM2_ESM.pdf]

Reviewers' comments:

Reviewer #1 (Remarks to the Author):

In their manuscript, the authors reconstituted the biosynthetic pathway for the fungal polyketide bikaverin in yeast. The pathway for bikaverin was not fully elucidated. By recoding the pathway in yeast, the authors were able to determine the minimal gene set necessary for production of bikaverin. In addition, they developed a GFP-fusion strategy to confirm that all genes were well expressed and translated into proteins efficiently. To optimize the yield of bikaverin, they used a promoter exchange strategy to alter expression of the genes in the bikaverin pathway. Finally, when they fused Bik2 and Bik3 lead to a large increase in bikaverin titer.

The paper is well written and easy to understand. The work is a nice demonstration of the benefits of reconstituting biosynthetic pathways in an orthogonal host. The results are interesting but not too surprising.

Specific comments:

• Bik2-Bik3 tethering improves channeling: No proof, could stabilize the enzymes or do something else
• On page 7 the authors state "toxicity of Bik1 expression". Is expression toxic?
• Supplementary Figure 2: mass spectrum is garbage. I can't see anything in it.
• Fig 2e is poorly labeled. What do the numbers represent at the bottom of each plate containing the cell dilutions?
• You state that previously bikaverin was produced by F. fujikoroi. How does the production of bikaverin in yeast compare to that by F. fujikoroi?
• To prove that you have bikaverin, you should really prove it using NMR.
• The Bik2-Bik3 fusion may not increase channeling. It could stabilize one or both proteins. The GFP tagging of the proteins is not necessarily a good measure of protein folding.
• Figure 4a has "Bikaverin Yield (mg/L)" as the label. Its not a yield … it's a titer.

Reviewer #2 (Remarks to the Author):

Boeke and coworkers demonstrate a metabolic engineering application of a fungal polyketide in S. cerevisiae, which is a commonly used chassis for fungal polyketides, but they note that to the best of their knowledge, no further examples of pathway engineering exist. The paper is thorough, using a bottom up strategy that identified the essential genes for the polyketide, bikaverin and employed GFP-tagging, promoter exchange, and, strikingly, an enzyme fusion method to achieve a 60-fold increase in yield.

The chimera of Bik2 and Bik3 was a somewhat unusual approach to take in this sort of system, and worked quite well which is an interesting scientific finding.

A few minor concerns:

In line 168 the authors claim that deletion of either ppt1 or npgA reduced bikaverin yield 2 fold compared to the original strain, however bikaverin was still successfully synthesized in both cases, so this suggests that both Ppt1 and NpgA are capable of activating the ACP domain of Bik1, but one copy of either gene is not enough to allow for full posttranslational modification. To me it seems like that experiment is not quite supported by that result. Is it possible that Ppt1 is more active on some ACP domains than NpgA, meaning it is more effectively phosphopantetheinylated? Without making a strain that has two copies of NpgA and a strain that has two copies of Ppt1 integrated, it seems to be impossible to make that claim. This is a minor point, and the paper should not hinge upon doing this experiment, but I still think the authors should revise the text to reflect this.

Line 231: I would like a more detailed discussion and/or characterization of “dead end” intermediates. What exactly are they?

Line 245: The discussion of the intermediate channel is not entirely clear and not clear to me from the homology model presented in the supplemental.

Finally, prior to publication, I think the authors should include full sequences of codon optimized genes used in this study and the fusion construct of Bik2-Bik3 in the supplemental. I think having this sort of information transparently present in the supplemental is pretty critical for enabling synthetic biology.

Reviewer #3 (Remarks to the Author):

Fungi harbor iterative PKS type I and produce a multitude of bioactive compounds. One of the best characterized fungal PKS is the NorS complex, which was analyzed in “deconstructive approach”. A homologous protein was recently characterized in the structure of its condensing part and loading unit. In spite of this knowledge, fungal PKS have so far just rarely been used for pathway engineering in yeast. Studies to the engineering of fungal PKS for the design of chimeric constructs are essentially non-existent.
Zhao et al describe the reconstruction of the biosynthetic pathway of the formation of bikaverin, a tetracyclic polyketide with antibiotic, antifungal and anticancer properties, in S. cerevisiae. The heart of this pathway is the iterative PKS Bik1, producing the polycyclic compound that is further turned over by the monooxygenase (Bik2) and methyltransferase (Bik3). This study essentially demonstrates that pathways for the synthesis of complex polyketides can be reconstituted in S. cerevisiae.

The are several interesting aspects in this manuscript, in addition to the overall successful endeavor to produce this compound, which makes the study in general very valuable. The manuscript is also well written with a good logical linking of experimental data. All in all the authors succeed in presenting a compelling narrative.

There is one critical aspect in this manuscript with large consequences. Conclusions drawn in this manuscript suffer from completely ignoring protein quality aspects. The genes of this pathway were deleted, exchanged and fused, and the success of these experiments was evaluated on the product level; i.e., the identity and the amount of the detected product. How about the proteins? There are especially two important aspects, where the properties of the protein could help in understanding data.
(i) GFP-fusions for monitoring protein concentration levels in the cells are not new, but nevertheless elegant. Its use to judge protein levels of a PKS, roughly 180 kDa in size, is very critical. Multidomain proteins are prone to proteolytic degradation and aggregation, and this approach does therefore not unambiguously allow checking protein levels when not proving protein quality before. There may just be GFP giving the signal, while the N-terminal protein of interest is degraded. Protein quality can be checked by Western Blot or more easily by immunoprecipitation with anti-GFP Nanobody followed by SDS-PAGE.
(ii) The effect of the gene fusion on proteins Bik2 and Bik3 is just poorly characterized. GFP levels give a poor estimate on the protein levels in the cell and do not allow to judge the quality of the protein in the cell. Bik2 or Bik3 may be proteolytically degraded or aggregating as separate protein, but stabilize each other upon fusion. Such an effect would be invisible in the GFP approach. A minimal solution could again be immunoprecipitation with anti-GFP Nanobody and SDS-PAGE. The pattern of band could at least give an impression of proteolytic quality. It should be noted the proximity channeling is extremely controversially discussed. Proximity can have direct effect on the kinetics just at high turnovers and close proximity. Both of these requirements are likely not fulfilled. Please see Zhang, Y. & Hess, H. T. (2007) ACS Catalysis.

Although the manuscript is generally well writing I strongly recommend toning down the manuscript in it is conclusions and impact for the field. In several of those aspect the manuscript is overselling. See, for example, paragraph lines 289-301:

(i) This study "greatly expands the toolbox for biosynthetic pathway engineering". I do not see how the toolbox is expanded. GFP fusion for checking protein production in intact cells has been performed before many times. Please consider the problematic aspect of completely unclear protein quality. Top-down approaches to delete genes and analyze products are standard when analyzing synthetic gene clusters. Promotor exchange is not new and neither is the fusion of proteins. Please consider the problematic aspect of completely unclear effect upon gene fusion.

(ii) "The present results now allow for the implementation of similar combinatory hybrid PKS experiments in S. cerevisiae." Why? This study does not help in judging whether domain swapping for combinatory hybrid PKS experiments will be possible in yeast. Iterative fungal PKS have been expressed in yeast before (see e.g. expression of MSAS in yeast by Jens Nielsen and coworkers (Biotechnol. Bioeng. (2007)). So already that data put into prospect that domain swapping may be possible.

Please also consider: To which extent PKS can be rationally engineered has been discussed before many times. The statement, "…type I iterative PKS enzyme works as an assembly line with each domain catalyzing a different function and this predictability of function implied that PKSs can be rationally engineered." Is not correct. Type I PKS can perform cryptic coding, difficult to understand and not controllable so far for engineering, but does not perform assembly line synthesis. Assembly line synthesis is done by modular PKS. Please adapt.

This is a valuable but not groundbreaking study, which unfortunately leaves key questions open. The manuscript would surely benefit from a few experiments directed on the characterization of the protein quality. Such experiments could decisively improve the understanding of the system. Particularly, in the light of the appraisal of this study in paving the way for chimeric PKS design. At least, there should be a more critical interpretation of data and a more moderate tone on its impact for future studies.

Minor points:

-The results on the knockout of ppt1 or npgA are interesting. How does a deletion of both ppt1 AND npgA behave? Is there an intrinsic PPT that could be responsible for Ppantylation?

-It is very hard to understand how a few mutations should account the very different results of V1-2007 and V2-2013 (including data to bottom-up strategy to build constructs with different combinations of bik1, bik2 and bik3). Any idea on the origin of this difference.

-line 107: this should be 2007

-line 256: finale

The line numbers mentioned in our answers all refer to the newly revised version.

Reviewers' comments:

Reviewer #1 (Remarks to the Author):

In their manuscript, the authors reconstituted the biosynthetic pathway for the fungal polyketide bikaverin in yeast. The pathway for bikaverin was not fully elucidated. By recoding the pathway in yeast, the authors were able to determine the minimal gene set necessary for production of bikaverin. In addition, they developed a GFP-fusion strategy to confirm that all genes were well expressed and translated into proteins efficiently. To optimize the yield of bikaverin, they used a promoter exchange strategy to alter expression of the genes in the bikaverin pathway. Finally, when they fused Bik2 and Bik3 lead to a large increase in bikaverin titer.

The paper is well written and easy to understand. The work is a nice demonstration of the benefits of reconstituting biosynthetic pathways in an orthogonal host. The results are interesting but not too surprising.

We appreciate your comments to improve this manuscript. We agree that using *Saccharomyces cerevisiae* to produce a complicated polyketide like bikaverin, which requires a typical iterative type I PKS and a long chain condensed by PKS iterative reactions, and to a high titer, is impressive. Our study demonstrated the use of synthetic biology approaches to transplant a complex type I polyketide pathway into *Saccharomyces cerevisiae* (Brewer's yeast), as well as troubleshooting and optimizing yields. Our study essentially demonstrated that pathways for the synthesis of complex polyketides can be reconstituted in S. cerevisiae with a high-efficiency. In the revised manuscript, we implemented several necessary experiments to check protein stability and the channeling effect. We further improved bikaverin titers to ~202.75 mg/L (5 fold higher than \ highest titer in the original manuscript).

Specific comments:

1. Bik2-Bik3 tethering improves channeling: No proof, could stabilize the enzymes or do

something else.

This question is related to Question 7. To check protein stability, we labeled Bik2, Bik3, Bik2-Bik3 with 6×His tag, and did immunoblots using anti-His antibody. For each of them, one single band was detected at the expected protein size, with no obvious degradation (supplementary Fig. 11). Expression levels are similar to each other. As a result, the improvement of bikaverin titer cannot be due to stabilization resulting from fusion of Bik2 and Bik3. We think Bik2-Bik3 fusion contributes to a steric effect, via an efficient substrate-shuttle channel between the Bik2 and Bik3, allowing substrates to channel efficiently between the Bik2 and Bik3 proteins, and contributing to improved bikaverin titer.

To further support the channeling hypothesis, we also made the "reverse" fusion protein, Bik3-Bik2. It was also stable, with similar expression. The bikaverin titer, however, was reduced dramatically. In the other words, the fusion "orientation" is critical, which strongly supports that Bik2-Bik3 tethering improves channeling.

2. On page 7 the authors state "toxicity of Bik1 expression". Is expression toxic?

It is a little bit toxic but not strong. Bik1 is a large protein with multiple domains, roughly 220 kDa in size. To check for toxicity, we performed a spot assay for the yeast strain carrying the plasmid of P_{GAL1}-*bik1* with *URA3* marker, on SC–Ura w/ galactose medium, as shown in Supplementary Fig 1. It grew similarly to the control empty vector. The strain also turned red, indicating that bikaverin was successfully produced, therefore Bik1 was successfully expressed in this experiment.

Nevertheless, expressing such a large exogenous protein may represent a burden for yeast. We did observe some of degradation of Bik1 in yeast, as shown in western blot (Fig.2d), although some of this degradation may occur during purification. Importantly a very substantial percentage of the protein appears to be full-length, consistent with the fact that we observe excellent production of the end product.

3. Supplementary Figure 2: mass spectrum is garbage. I can't see anything in it.

This figure shows initial mass spectrum data confirming production of bikaverin, which is also addressed in Supplementary Fig. 4 (as the bikaverin standard) and Supplementary Fig. 8f (as the experiment result). We also realized that the resolution of original version was unsuitable for publication so we reorganized the data and prepared a new Supplementary Fig. 2

4. Fig 2e is poorly labeled. What do the numbers represent at the bottom of each plate containing the cell dilutions?

The numbers refer to the concentration of estradiol (EST) added into the media. This figure is used for confirming the effect of Bik1's expression on bikaverin synthesis. Strains growing on the plate with higher EST concentrations will express Bik1 at a higher level. We added the necessary labels to make this figure clear. In the revised manuscript, the original Fig. 2e is the new Fig. 3b.

5. You state that previously bikaverin was produced by F. fujikoroi. How does the production of bikaverin in yeast compare to that by F. fujikoroi?

Different strains of *F. fujikuroi* demonstrate a wide variety of bikaverin production capacities. To our knowledge, some wild *F. fujikuroi* strains have been reported to produce bikaverin ranging from 25 mg/L to 3 g/L (*F. fujikuroi P-3* with 25 mg/L, *F. fujikuroi* U with 458 mg/L, *F. fujikuroi* (Sawada) CDBB H-984 with 3 g/L) (references listed below). In 2016, G. J. Lale screened a mutant strain with a maximal 6.3 g/L bikaverin production after optimizing the C: N ratio in medium. In our study, *S. cerevisiae* produced 202 mg/L bikaverin. Although the titer in *S. cerevisiae* is still lower than the highest one in *F. fujikuroi,* it is close to some wild strains of *F. fujikuroi*, showing the potential to efficiently produce complex fungal polyketides. Given much more available genetic tools and the ease of cell engineering with *S. cerevisiae*, further optimization is possible. So we think yeast is still a better test system for mining, studying and engineering fungal polyketides.

References used here:

Kumar PK, Losane BK. “Immobilized growing cells of Gibberella fujikuroi P-3 for the production gibberellic acid and pigment in batch and semi-continuous cultures.” Appl

Microbiol Biotechnol(1988)28:537–42.

Balan J, Fuska J, et al. “Bikaverin, an antibiotic from Gibberella fujikuroi effective against Leishmania brasiliensis. ”Folia Microbiol(1970)15:479–83.

Escamilla-Silva E, Poggi-Varaldo H, et al. “Selective production of bikaverin in fluidized bioreactor with immobilized Gibberella fujikuroi.” World J Microbiol Biotechnol(2001)17:469–74.

G. J. Lale and R. V. Gadre. “Production of bikaverin by a Fusarium fujikuroi mutant in submerged cultures.” AMB Express (2016) 6:34

6. To prove that you have bikaverin, you should really prove it using NMR.

Bikaverin is not a novel chemical. Its structure and absorbance have been well studied. We have confirmed its production by HPLC-Mass Spec and UV Spec. We also compared those data with that of commercially available bikaverin (Sigma, SML0724) as the standard, which was not detected in the negative control yeast strains with empty vector. We believe this is sufficient chemical evidence confirming production of bikaverin.

7. The Bik2-Bik3 fusion may not increase channeling. It could stabilize one or both proteins. The GFP tagging of the proteins is not necessarily a good measure of protein folding.

This question is related to question 1. To further confirm stability, we labeled Bik2, Bik3, Bik2-Bik3 with the poly-His tags, and performed immunoblotting. It clearly showed that Bik2, Bik3 and Bik2-Bik3 were stable with no degradation, as in Supplementary Fig. 11.

8. Figure 4a has “Bikaverin Yield (mg/L)” as the label. Its not a yield … it's a titer.

Great point. Indeed “(mg/L)” is not a unit of yield. The “titer” is a more appropriate expression for our case. We also checked the full manuscript and used the correct expression.

Reviewer #2 (Remarks to the Author):

Boeke and coworkers demonstrate a metabolic engineering application of a fungal polyketide in S. cerevisiae, which is a commonly used chassis for fungal polyketides, but they note that to the best of their knowledge, no further examples of pathway engineering exist. The paper is thorough, using a bottom up strategy that identified the essential genes for the polyketide, bikaverin and employed GFP-tagging, promoter exchange, and, strikingly, an enzyme fusion method to achieve a 60-fold increase in yield.

The chimera of Bik2 and Bik3 was a somewhat unusual approach to take in this sort of system, and worked quite well which is an interesting scientific finding.

Thanks a lot for your review, suggestions and positive feedbacks.

A few minor concerns:

1. In line 168 the authors claim that deletion of either ppt1 or npgA reduced bikaverin yield 2 fold compared to the original strain, however bikaverin was still successfully synthesized in both cases, so this suggests that both Ppt1 and NpgA are capable of activating the ACP domain of Bik1, but one copy of either gene is not enough to allow for full posttranslational modification. To me it seems like that experiment is not quite supported by that result. Is it possible that Ppt1 is more active on some ACP domains than NpgA, meaning it is more effectively phosphopantetheinylated? Without making a strain that has two copies of NpgA and a strain that has two copies of Ppt1 integrated, it seems to be impossible to make that claim. This is a minor point, and the paper should not hinge upon doing this experiment, but I still think the authors should revise the text to reflect this.

This is an interesting question. To answer that, we performed the experiment to test bikaverin production from the strains containing two copies of Ppt1 or NpgA, as in Supplementary Fig. 3. Interestingly, compared to the single copy of Ppt1, doubling Ppt1 didn’t lead to an increase of bikaverin titer, which was still around half of single copy of Ppt1+NgpA.

But for NpgA, two copies of that did improve the bikaverin titer to the similar level of original strains containing Ppt1+NpgA. This indicates to us that NpgA was actually more effective than Ppt1 in activating the ACP domain of Bik1 in yeast.

As generic method, we still prefer the design of Ppt1+NpgA. First, it is a safe initial design to activate ACP domains before any tests. Ppt1+NpgA was also more stable compared to two copies of NpgA in yeast, where there is a strong homologous recombination ability.

As the reviewer said, this is a minor point. So we modified our statements as in line 148-158, and deleted the statement "one copy of either gene did not provide enough catalytic potential to allow for post-translational activation of the produced Bik1 enzyme."

2. Line 231: I would like a more detailed discussion and/or characterization of "dead end" intermediates. What exactly are they?

This about Fig. 4. Originally, we referred to C8-me-pre-bikaverin (3) and C3-me-pre-bikaverin (4). Pre-bikaverin was first oxidized by Bik2 to form oxo-pre-bikaverin. So these two intermediates (3, 4) were not a part of major reactions, thus we called them "dead end".

We also feel that this description is not clear and may be misleading. So, we deleted this expression to avoid any confusion.

3. Line 245: The discussion of the intermediate channel is not entirely clear and not clear to me from the homology model presented in the supplemental.

The line 245 in the original submitted manuscript is the very last line in the result section, right before the discussion section.

We also realized the original description is unclear and confusing. And we re-edited this part as in line 212-240. In our paper, homology model was used for supporting the fusion direction. It not an evidence for showing the channel. So we put it in the supplementary materials (Supplementary Fig. 10).

In our study, the Bik2-Bik3 fusion protein dramatically improved titer of bikaverin. Through western blot, we found Bik2, Bik3, Bik2-Bik3 were all stable with no obvious degradations (Supplementary Fig. 11). Their expression levels were also close with no significant difference. We also built the "reverse" fusion protein, as Bik3-Bik2, which was also stable with the same expression level. The bikaverin titer, however, was reduced dramatically. We concluded that the improvement of bikaverin titer was not due to the stabilization from the fusion of Bik2 and Bik3. Based on this, we believe that the Bik2-Bik3 fusion contributes a steric effect, namely an efficient substrate-shuttle channel between the Bik2 and Bik3, allowing substrates to channel efficiently between Bik2 and Bik3, and ultimately contributing to improved bikaverin titer.

4. Finally, prior to publication, I think the authors should include full sequences of codon optimized genes used in this study and the fusion construct of Bik2-Bik3 in the supplemental. I think having this sort of information transparently present in the supplemental is pretty critical for enabling synthetic biology.

We are pleased to include the full DNA sequence, especially if this is critical for enabling other synthetic biology studies. The DNA sequences of codon optimized *bik1*, *bik2*, *bik3*, *bik6*, *ppt1*, *npgA*, *bik2-bik3* and *bik3-bik2* were listed and labelled in Supplementary Tale 4.

Reviewer #3 (Remarks to the Author):

Fungi harbor iterative PKS type I and produce a multitude of bioactive compounds. One of the best characterized fungal PKS is the NorS complex, which was analyzed in "deconstructive approach". A homologous protein was recently characterized in the structure of its condensing part and loading unit. In spite of this knowledge, fungal PKS have so far just rarely been used for pathway engineering in yeast. Studies to the engineering of fungal PKS for the design of chimeric constructs are essentially non-existent.

Zhao et al describe the reconstruction of the biosynthetic pathway of the formation of bikaverin, a tetracyclic polyketide with antibiotic, antifungal and anticancer properties, in S. cerevisiae. The heart of this pathway is the iterative PKS Bik1, producing the polycyclic

compound that is further turned over by the monooxygenase (Bik2) and methyltransferase (Bik3). This study essentially demonstrates that pathways for the synthesis of complex polyketides can be reconstituted in S. cerevisiae.

The are several interesting aspects in this manuscript, in addition to the overall successful endeavor to produce this compound, which makes the study in general very valuable. The manuscript is also well written with a good logical linking of experimental data. All in all the authors succeed in presenting a compelling narrative.

There is one critical aspect in this manuscript with large consequences. Conclusions drawn in this manuscript suffer from completely ignoring protein quality aspects. The genes of this pathway were deleted, exchanged and fused, and the success of these experiments was evaluated on the product level; i.e., the identity and the amount of the detected product. How about the proteins? There are especially two important aspects, where the properties of the protein could help in understanding data.

1. GFP-fusions for monitoring protein concentration levels in the cells are not new, but nevertheless elegant. Its use to judge protein levels of a PKS, roughly 180 kDa in size, is very critical. Multidomain proteins are prone to proteolytic degradation and aggregation, and this approach does therefore not unambiguously allow checking protein levels when not proving protein quality before. There may just be GFP giving the signal, while the N-terminal protein of interest is degraded. Protein quality can be checked by Western Blot or more easily by immunoprecipitation with anti-GFP Nanobody followed by SDS-PAGE.

We checked protein quality with of a 6xHis-tagged Bik1 protein on a western blot as in Fig. 2d. and we found that Bik1 indeed showed some level of protein degradation; however, a strong band was observed that corresponds to full -length protein. But, when driven by the strong *GAL1* promoter, Bik1 was highly expressed and the functional Bik1 clearly completes the first step of bikaverin synthesis effectively.

We cannot rule out the possibility that this degradation happened during the protein sample preparation for the western blot, especially for this large PKS protein (221 kD). But nevertheless, functional Bik1 was successfully expressed in yeast.

2. The effect of the gene fusion on proteins Bik2 and Bik3 is just poorly characterized. GFP levels give a poor estimate on the protein levels in the cell and do not allow to judge the quality of the protein in the cell. Bik2 or Bik3 may be proteolytically degraded or aggregating as separate protein, but stabilize each other upon fusion. Such an effect would be invisible in the GFP approach. A minimal solution could again be immunoprecipitation with anti-GFP Nanobody and SDS-PAGE. The pattern of band could at least give an impression of proteolytic quality. It should be noted the proximity channeling is extremely controversially discussed. Proximity can have direct effect on the kinetics just at high turnovers and close proximity. Both of these requirements are likely not fulfilled. Please see Zhang, Y. & Hess, H. T. (2007) ACS Catalysis.

To address this question, we relabeled Bik2, Bik3, Bik2-Bik3 with the short 6xHis tag, and performed a western blot using anti-His tag antibody. As shown in Supplementary Fig. 11, Bik2, Bik3 and their fusion proteins have no detectable degradation and similar expression levels. We further tried the “reverse” fusion. The reverse Bik3-Bik2 fusion protein was also stable with no degradation and the same expression level as Bik2-Bik3. But in the case of the reverse fusion, bikaverin production was dramatically reduced compared to Bik2-Bik3. We concluded that the improvement of bikaverin titer was not due to the stabilization from the fusion of Bik2 and Bik3.

We speculated that the Bik2-Bik3 fusion contributes to a steric effect, that may form an efficient substrate-shuttle channel between Bik2 and Bik3 active sites, allowing substrates to channel efficiently between the Bik2 and Bik3, thereby contributing to an improved bikaverin titer. This question was also asked by Reviewer 1, as in Questions 1 and 7.

We understand that proximity channeling is potnetially controversial; but we emphasize the fact that this is a hypothesis. Mechanistically proving this is beyond the scope of this manuscript. Here, we hope to discuss about our speculation based on our fusion protein result. We also toned down our statements about this part, as in line 280-286 now.

3. Although the manuscript is generally well writing I strongly recommend toning down the manuscript in it is conclusions and impact for the field. In several of those aspect the manuscript is overselling. See, for example, paragraph lines 289-301: This study “greatly expands the toolbox for biosynthetic pathway engineering”. I do not see how the toolbox is expanded. GFP fusion for checking protein production in intact cells has been performed before many times. Please consider the problematic aspect of completely unclear protein

quality. Top-down approaches to delete genes and analyze products are standard when analyzing synthetic gene clusters. Promotor exchange is not new and neither is the fusion of proteins. Please consider the problematic aspect of completely unclear effect upon gene fusion.

GFP tag method is elegant and easy to modify/edit/check synthesis pathways in practice. In our case, the GFP-Mapping helped us identify the bottleneck of Bik-pathway and the protein fusion strategy also worked quite well in our system. But at the reviewer's request, we toned down our statements.

In the original manuscript, line 289 was the last paragraph of the discussion section. In the revision, we deleted "greatly expands…engineering", and add new statements as "By establishing and optimizing the bikaverin pathway, we have demonstrated effective and practical methods for the metabolic engineering of polyketides in yeast", as in line 271.

4. "The present results now allow for the implementation of similar combinatory hybrid PKS experiments in S. cerevisiae." Why? This study does not help in judging whether domain swapping for combinatory hybrid PKS experiments will be possible in yeast. Iterative fungal PKS have been expressed in yeast before (see e.g. expression of MSAS in yeast by Jens Nielsen and coworkers (Biotechnol. Bioeng. (2007)). So already that data put into prospect that domain swapping may be possible.

PKS enzyme engineering is difficult but attractive. We also look forward to breakthroughs in this direction, especially for generating new PKS enzymes and polyketide derivatives. Previously, functional MSAS was expressed in yeast, to synthesize the polyketide 6-MSA. In our study, we successfully expressed another PKS (Bik1) and biosynthesizes bikaverin, which is more complicated structurally than 6-MSA. Bikaverin, and other similar tetracyclic chemicals may have more potentials for scientists to discover novel bioactive derivatives.

We didn't test domain swapping in Bik1 in this study, which seems beyond the scope of this project. We hope to make our contribution to this field by building the platform containing functional PKS and demonstrate the downstream modification process.

Here, based on reviewer's suggestion, we re-wrote the last paragraph in the discussion

section. The new statement is “With more and more PKS successfully expressed in yeast, we anticipate the expansion of metabolic engineering of PKS in S. cerevisiae, including the development of methods in yeasto, such as domain swapping to generate diverse polyketides. It has been reported that swapping domains among different PKSs results in novel diverse functions in vitro.”, as in line 288.

5. Please also consider: To which extent PKS can be rationally engineered has been discussed before many times. The statement, “…type I iterative PKS enzyme works as an assembly line with each domain catalyzing a different function and this predictability of function implied that PKSs can be rationally engineered.” Is not correct. Type I PKS can perform cryptic coding, difficult to understand and not controllable so far for engineering, but does not perform assembly line synthesis. Assembly line synthesis is done by modular PKS. Please adapt.

Thanks for your checking and careful thinking. This was also the last paragraph in the discussion section. Since PKS enzyme engineering is not the major part of this study, we removed this statement to avoid any confusion.

6. This is a valuable but not groundbreaking study, which unfortunately leaves key questions open. The manuscript would surely benefit from a few experiments directed on the characterization of the protein quality. Such experiments could decisively improve the understanding of the system. Particularly, in the light of the appraisal of this study in paving the way for chimeric PKS design. At least, there should be a more critical interpretation of data and a more moderate tone on its impact for future studies.

Thanks a lot for your reading and careful thinking. These comments and suggested experiments helped to make this manuscript better. In the revised version, we checked protein quality using western blots and found that Bik2, Bik3 and their fusion proteins (Bik2-Bik3, Bik3-Bik2) were all in good quality with similar expression level. Besides, the “opposite polarity” fusion Bik3-Bik2 was much less efficient than the Bik2-Bik3 fusion in terms of production of bikaverin, which is strongly support for channeling effect. We also used a systematic GFP-Mapping strategy to easily detect the bottle neck step and continuously improved the bikaverin titer by promoter swapping. Moreover, we have successfully improved the titer to around 273 fold compared to the original construct, demonstrating

significant potential for *S. cerevisiae* to product complex polyketides.

We will be very pleased if this study can benefit future research on chimeric PKS design and engineering. In revised manuscript, we also toned down the statements about this part.

Minor points:

-The results on the knockout of ppt1 or npgA are interesting. How does a deletion of both ppt1 AND npgA behave? Is there an intrinsic PPT that could be responsible for Ppantylation?

There is no intrinsic PPTase in yeast that can activate the ACP domain of this PKS. We performed experiment of deleting both ppt1 and npgA to prove it, as in line 148-150. This was also studied before that an exogenous PPTase is required (Wattanachaisaereekul, Songsak, *et al*. Biotechnology and bioengineering 97.4 (2007): 893-900).

-It is very hard to understand how a few mutations should account the very different results of V1-2007 and V2-2013 (including data to bottom-up strategy to build constructs with different combinations of bik1, bik2 and bik3). Any idea on the origin of this difference.

The difference was shown as Supplementary Fig. 1, in the original manuscript. We checked the sequence, and found the mutations of Bik1 map to its SAT domain. The mutations in Bik2 map to the Rossmann-fold NAD(P)(+)-binding region. These mutations may lie in active regions of proteins or affect their folding.

We realized that this is a minor point of this manuscript but it caused much more confusion. So we have removed this part of the revised manuscript.

-line 107: this should be 2007

The original sentence has been deleted, as explained above.

-line 256: finale

This typo has been corrected.

Reviewer #1 (Remarks to the Author):

The revised manuscript is significantly improved over the previous version of the manuscript. They substantially improved the Bikavirin titer and they provided more supporting evidence for many of the claims that were suspect in the previous version. Some minor comments:

1. I am still not fully convinced of the channeling argument, but I think this is a minor point.

2. In Figure 2f, the retention time of a peak in the sample from the engineered strain certainly matches the standard, but it would be good if the authors showed a trace of the parental strain with that peak missing.

Reviewer #2 (Remarks to the Author):

The revisions made to the manuscript satisfied my concerns. I am particularly intrigued they did the experiments with two copies of Ppt1 and NpgA.

Reviewer #3 (Remarks to the Author):

All my concerns have been addressed. Publish as is; no additional revisions needed.

Btw, the degradation band in the Western Blot for Bik1 may indicate proteolytic cleavage in the ACP-TE/CLC (or PT-ACP) linker. A further improvement in titer may be possible by modifying linker(s) in order to make them less degradation prone.

Here are the reviewers' comments and our responses during the second round revision. All our responses are highlighted in red. All the line numbers mentioned here are from the revised manuscript file newly submitted.

REVIEWERS' COMMENTS

Reviewer #1 (Remarks to the Author):

The revised manuscript is significantly improved over the previous version of the manuscript. They substantially improved the Bikaverin titer and they provided more supporting evidence for many of the claims that were suspect in the previous version. Some minor comments:

1. I am still not fully convinced of the channeling argument, but I think this is a minor point.

Thanks for your reviewing and comments that helped us improve this manuscript. As in the first round revision, we toned down our statements.

2. In Figure 2f, the retention time of a peak in the sample from the engineered strain certainly matches the standard, but it would be good if the authors showed a trace of the parental strain with that peak missing.

We do have the data of parental strain carrying empty vector from the same original experiment. As requested, figure 2f was updated to include this control (BY4742 + pRS416), in which the bikaverin peak was not detectable.

Reviewer #2 (Remarks to the Author):

The revisions made to the manuscript satisfied my concerns. I am particularly intrigued they did the experiments with two copies of Ppt1 and NpgA.

Thanks for your suggestions that helped us improve this manuscript with new experiments.

Reviewer #3 (Remarks to the Author):

All my concerns have been addressed. Publish as is; no additional revisions needed.
Btw, the degradation band in the Western Blot for Bik1 may indicate proteolytic cleavage in the ACP-TE/CLC (or PT-ACP) linker. A further improvement in titer may be possible by modifying linker(s) in order to make them less degradation prone.

Thanks for your comments and suggestions. We are happy to test this after this manuscript.
